# Reverse chemical ecology in a moth: machine learning on odorant receptors identifies new behaviorally active agonists

**DOI:** 10.1007/s00018-021-03919-2

**Published:** 2021-08-27

**Authors:** Gabriela Caballero-Vidal, Cédric Bouysset, Jérémy Gévar, Hayat Mbouzid, Céline Nara, Julie Delaroche, Jérôme Golebiowski, Nicolas Montagné, Sébastien Fiorucci, Emmanuelle Jacquin-Joly

**Affiliations:** 1 INRAE, Sorbonne Université, CNRS, IRD, UPEC, Université de Paris, Institute of Ecology and Environmental Sciences of Paris, 78000 Versailles, France; 2 Université Côte d’Azur, CNRS, Institut de Chimie de Nice UMR7272, 28 avenue Valrose, 06108 Nice, France; 3 Department of Brain and Cognitive Sciences, Daegu Gyeongbuk Institute of Science and Technology, Daegu, 711-873 South Korea; 4 Present Address: Disease Vector Group, Chemical Ecology Unit, Department of Plant Protection Biology, Swedish University of Agricultural Sciences, Alnarp, Sweden; 5 Present Address: Max Planck Centre Next Generation Chemical Ecology, Uppsala, Sweden

**Keywords:** Semiochemicals, Insects, *Spodoptera littoralis*, Behavior, Crop protection, Machine learning

## Abstract

The concept of reverse chemical ecology (exploitation of molecular knowledge for chemical ecology) has recently emerged in conservation biology and human health. Here, we extend this concept to crop protection. Targeting odorant receptors from a crop pest insect, the noctuid moth *Spodoptera littoralis*, we demonstrate that reverse chemical ecology has the potential to accelerate the discovery of novel crop pest insect attractants and repellents. Using machine learning, we first predicted novel natural ligands for two odorant receptors, SlitOR24 and 25. Then, electrophysiological validation proved in silico predictions to be highly sensitive, as 93% and 67% of predicted agonists triggered a response in *Drosophila* olfactory neurons expressing SlitOR24 and SlitOR25, respectively, despite a lack of specificity. Last, when tested in Y-maze behavioral assays, the most active novel ligands of the receptors were attractive to caterpillars. This work provides a template for rational design of new eco-friendly semiochemicals to manage crop pest populations.

**Supplementary Information:**

The online version contains supplementary material available at 10.1007/s00018-021-03919-2.

## Introduction

Insects detect and use odorant information from the external environment to make important decisions, such as selecting a mating partner, a food source or an oviposition site [1]. Depending on the species ecology, odorant signals can repel or attract insects, or do nothing. Among behaviorally relevant molecules, one can cite the moth sex pheromones that attract males from some distance away. Because of such olfactory-triggered behaviors, odorant molecules have been exploited to develop control strategies against insect pests and disease vector populations [2–4] that are integrated in combination with other strategies in integrated pest management. For instance, synthetic moth sex pheromones have been used for decades for population monitoring or mating disruption [2], aggregation pheromones and/or host plant volatiles are used for mass trapping, and non-host or toxic odorants are used as repellents. However, the identification of such active molecules is usually difficult, because it mainly relies on bioassay-guided approaches, including fastidious behavioral assays on multiple individuals.

In this context, reverse chemical ecology has recently emerged as a powerful alternative to identify relevant signals for a given species. This approach proposes to screen olfactory proteins linked to a particular behavior in order to identify putative behaviorally active semiochemicals [5]. It has been promoted by the recent advances in our understanding of the molecular basis of insect olfaction in the last two decades, especially the discovery of their odorant receptors (ORs) [6–8]. These ORs are transmembrane proteins primarily responsible for odorant detection. They are expressed in olfactory sensory neurons (OSNs) housed in olfactory sensilla, located mainly on the antennae. ORs form ion channels together with a subunit called Orco (OR coreceptor) that is highly conserved across insect species [9–11]. Odorants activate the corresponding OR-Orco complex that transforms the chemical signal into an electrical signal that is transmitted to the brain, leading to the behavioral response [12]. Identifying molecules that will be active on target ORs remains difficult [4], but ligand-based in silico strategies relying on the chemical structures of active compounds have proven quite effective for virtual screening of ORs. Quantitative-structure–activity relationship (QSAR) models, which have been widely used in medical chemistry [13, 14], have been applied with success to predict the activity of semiochemicals on ORs from model insects such as *Drosophila melanogaster* [15] and the mosquitoes *Aedes aegypti* and *Anopheles gambiae* [16–19].

In the present study, we used QSAR models to predict ligands for ORs from a non-model insect species, the crop pest moth *Spodoptera littoralis*, revealing it is possible to use machine learning to identify OR agonists outside Diptera [20]. We have previously identified ligands for a large number of *S. littoralis* ORs (hereafter SlitOR) using heterologous expression in the empty neuron system [21]. Moreover, behavioral assays have shown that *S. littoralis* caterpillars are attracted by plant volatiles that activate SlitOR24 and SlitOR25 [22]. With the final aim of identifying new attractive semiochemicals for *S. littoralis* larvae, we thus prioritized these two ORs that presented a large overlapping receptive range, including aromatic compounds and green leaf volatiles. We virtually screened a judiciously selected natural product library to identify novel ligands. This led to success rates of 67% and—even more impressively—93% active molecules on SlitOR25 and SlitOR24, respectively. Finally, we conducted behavioral experiments to investigate the activity of the most potent agonists of SlitOR24 and SlitOR25. This work, combining machine learning, electrophysiological analyses and behavioral assays, not only expands the list of natural SlitOR ligands but also successfully identifies new larval attractants that can potentially be implemented in eco-friendly control strategies. Whereas the concept of reverse chemical ecology has been successfully applied in conservation biology (targeting endangered species [23]) and human health (targeting disease vectors [5]), our work now demonstrates its great potential in agriculture.

## Materials and methods

### Insects

*S. littoralis* larvae were reared on a semi-artificial diet [24] under the following conditions: 22 °C, 60% relative humidity and 16:8-h light: dark cycle. Fourth-instar larvae (L4) were used for behavioral assays.

Transgenic *D. melanogaster* flies expressing SlitOR24 and 25 were obtained by crossing the lines *w;Δhalo/CyO;UAS-SlitOR24* and *w;Δhalo/CyO;UAS-SlitOR25* [21] with the line *w;Δhalo/CyO;OR22a-Gal4* [25]. Flies were reared on standard nutrient medium made of cornmeal, yeast and agar. Flies were kept at 25 °C, under a 12:12-h light: dark cycle.

### Modeling

#### Datasets

The SlitOR24 QSAR model was built using the dataset of 51 experimentally tested molecules (10 actives, 41 inactives) from [21]. The SlitOR25 model was built using the same dataset enriched with 32 molecules experimentally tested in [20], resulting in a dataset of 83 molecules labeled as active (25 molecules) or inactive (58 molecules) against SlitOR25. An in-house library of 158 plant volatile organic compounds (Online Resource 1) was screened by the two numerical models. All molecules were collected as SMILES strings, the major tautomers at pH 7.0 were retrieved with cxcalc (Calculator Plugins, Marvin 18.3.0, 2018, ChemAxon), and the resulting molecules were standardized with the standardizer python package v0.1.7 (for salt removal and structure normalization). Molecular descriptors were computed directly from the standardized SMILES using Dragon v6.0.38. Feature exclusion was performed within the software based on the following criteria: constant or near-constant descriptors, descriptors with at least one missing value and highly correlated descriptors (absolute pair correlation greater than or equal to 0.95 for SlitOR25 and 0.9 for SlitOR24) were excluded. This resulted in libraries of 288 and 493 descriptors for SlitOR24 and SlitOR25, respectively.

The SlitOR24 and SlitOR25 datasets (Online Resource 2) were split in training and test sets using a common clustering method, the sphere-exclusion approach, which can select a diverse subset of compounds in a dataset. For both sets, descriptors were normalized between 0 and 1, and the split was initialized by putting in the test set the compound closest to the center of the normalized dataset. At each iteration the new compound to be added to the test set was selected using a MinMax procedure, the dissimilarity radius to exclude compounds from the test set was set to 4.8 for SlitOR24 and 4.0 for SlitOR25, and the algorithm was stopped once the test set reached 24% of the size of the original dataset. This resulted in training sets of 39 molecules (8 actives, 31 inactives) and 64 molecules (18 actives, 46 inactives), and test sets of 12 molecules (two actives, 10 inactives) and 19 molecules (seven actives, 12 inactives) for SlitOR24 and SlitOR25, respectively. For both datasets, each descriptor was then denormalized and normalized only based on the training set min and max values. To quantify the uncertainty of prediction resulting from the initial choice of compounds in the training and test sets, five alternative splits were generated using the same strategy. The same sphere-exclusion approach was used to define the new training/test sets with initial compounds chosen randomly and not at the center of the normalized distribution as for the final model. Due to imbalanced data (less active than inactive compounds) and to facilitate comparison, only the first five splits that had the same activity distribution (active/inactive) as in the split used for the final model were investigated.

#### Machine-learning

QSAR models were trained and evaluated using Weka v3.8.2 [26]. Several classification algorithms were optimized in “leave-one-out” (LOO) cross-validation loops: C-SVC (LibSVM v1.0.10) (SVC: Support Vector Classifier; SVM: Support Vector Machine), k-nearest neighbors (kNN), RandomTree, DecisionTree, and RandomForest. Cost-sensitive models were also tested without providing a significant improvement in performance. Once the optimal algorithm and hyperparameters were identified for each OR based on Matthews correlation coefficient (MCC) (Table 1), the final models were trained on the full training set and parametrized as follow: for SlitOR24 a RandomForest was chosen and trained with 100 trees, unlimited maximum depth for each tree, and no feature randomly chosen; for SlitOR25 a kNN classifier (IBk) was chosen with nine neighbors, weighted by the inverse of the Euclidean distance, and a brute force neighbor search. Finally, the performances of the SlitOR24 and SlitOR25 models were assessed on the test sets.

**Table 1:** Performance evaluation of the SlitOR24 and SlitOR25 QSAR models using different metrics

	Dataset	TP	TN	FP	FN	Accuracy	Precision	Recall	FPR	MCC	AUROC
SlitOR24	LOO	4	29	2	4	0.85	0.67	0.50	0.06	0.49	0.83
	Training	7	31	0	1	0.97	1.00	0.88	0.00	0.92	0.99
	Test	1	9	1	1	0.83	0.50	0.50	0.10	0.40	0.80
SlitOR25	LOO	15	34	12	3	0.77	0.56	0.83	0.26	0.52	0.84
	Training	18	46	0	0	1.00	1.00	1.00	0.00	1.00	1.00
	Test	5	10	2	2	0.79	0.71	0.71	0.17	0.55	0.89

*LOO* performance of the best model using a leave-one-out cross-validation strategy, *TP* true positives, *TN* true negatives, *FP* false positives, *FN* false negatives, *FPR* false positive rate, *MCC* Matthews correlation coefficient, *AUROC* area under the receiver-operating characteristics curve

#### Applicability domain

A similarity distance approach [27] was used to estimate the applicability domain of the two selected models. A distance cutoff is defined as $Dc=\langle D\rangle +Z\sigma$ where $\langle D\rangle$ and $\sigma$ are the mean and standard deviation of Euclidean distances of each training set compound with their nearest neighbor in the descriptor space, and *Z* is an empirical parameter. The parameter *Z* was incremented until all training set compounds had their distance with their kNN lower or equal to *Dc*. For SlitOR25, we kept the same number of neighbors as in the model (*k* = 9) and for SlitOR24, we used *k* = 6 based on our benchmark of different learners during the training phase. For each external compound, its distance with the kNN was measured and a reliability score was estimated as $reliability=1+\frac{D-Dc}{Dc}$.

### Single sensillum recordings on neurons expressing SlitOR24 and SlitOR25

Single sensillum recordings were performed on *Drosophila* ab3A neurons expressing SlitOR24 or SlitOR25, using fly lines previously generated [21]. A 2–8-day-old fly was immobilized in a pipette tip, only the head sticking out. The fly was placed on a microscope glass slide under a constant 1.5 L min^−1^ flux of charcoal-filtered and humidified air delivered through a glass tube of a 7 mm diameter. The experiments were monitored using a light microscope (Olympus BX51WI, Tokyo, Japan) equipped with a × 100 magnification objective. Action potentials from ab3A OSNs were recorded using electrolytically sharpened tungsten electrodes (TW5-6, Science Products, Hofheim, Germany). One reference electrode was inserted into the eye and the recording electrode was inserted at the base of an ab3 sensillum using a motor-controlled PatchStar micromanipulator (Scientifica, Uckfield, United Kingdom). Odorants were purchased from Sigma-Aldrich (Saint-Louis, MO, USA). Stimulus cartridges were built by placing a 1 cm^2^ filter paper in a Pasteur pipette and loading 10 μL of the odorant solution onto the paper (10^–2^ dilution in paraffin oil), or 10 μL of paraffin oil or a paper without any odorant as controls. Odorant stimulations were performed by inserting the tip of the pipette into a hole in the glass tube and generating a 500 ms air pulse (0.6 L min^−1^). The responses of ab3A OSNs were calculated by subtracting the spontaneous firing rate (in spikes.s^−1^) from the firing rate during the odorant stimulation.

The stimulation panel consisted, for each SlitOR, of an already known agonist [21] used as positive control (benzyl alcohol for SlitOR24 and acetophenone for SlitOR25), paraffin oil as a negative control, 34 predicted agonists and 5 molecules randomly chosen among the predicted non-agonists for both ORs (Online Resource 3). Each stimulus cartridge was used at maximum eight times in total. The panel of molecules was tested on five (for predicted non-agonists) to eight-ten (for predicted agonists) different flies expressing SlitOR24 or SlitOR25. Odorants were considered as active if the response was statistically different from the response elicited by the solvent alone (Kruskal–Wallis test followed by a Dunnett multiple comparison test, *p* < 0.05).

### Larvae behavior in Y-tube olfactometer

Behavioral experiments were performed in a Y-tube olfactometer. The olfactometer consisted of a 2.1 cm inner diameter glass Y-tube, the main segment was 13 cm long, and each of the two arms was 9.5 cm long. L4 larvae were used and starved overnight (16–20 h starvation) prior to the experiments. All experiments were performed under red light, to avoid biases due to visual cues. Charcoal-purified air was delivered into each arm of the olfactometer at a flow rate of 0.5 L min^−1^, stabilized using a flowmeter (Key Instruments, Trevose, PA, USA) to ensure that equal air streams entered each arm. The temperature of the room was maintained at 24 °C during all tests. The experimental set-up was first tested with different controls: (1) paraffin oil in each arm, a configuration expected to induce no larval choice, (2) a 10^–2^ dilution of benzyl alcohol, an odorant known to induce larvae attraction [22], in one arm and paraffin oil in the other arm (larval choice expected) and (3) a 10^–2^ dilution of (*E*)-ocimene, a molecule inactive on larval behavior [22], versus paraffin oil (no larval choice expected). Seven of the strongest agonists of both SlitOR24 and 25 were tested for behavioral activity. Odorants were diluted in paraffin oil (dilutions 10^–2^ and 10^–3^). Ten µL of diluted odorants or control (paraffin oil) were loaded on a filter paper. A paper with solvent alone was placed in one arm and a filter paper with the odorant dilution in the other arm. One larva at a time was placed in the main arm of the olfactometer and the behavior was recorded during 10 min with a digital camera located above the device. Each larva was tested only once. To avoid any bias during the test, the olfactometer was switched from one side to the other between each test and up to three times, before washing the olfactometer with TFD4 detergent (Franklab, Montigny-le-Bretonneux, France) diluted at 3% for 30 min, then rinsing with distilled water and 95% ethanol. Once dry, all glass parts were put in an oven at 200 °C overnight. We analyzed two different parameters: (1) the choice made by the caterpillar and (2) the time spent in each arm. We considered that the caterpillar made a choice when three quarters of its body length entered an arm. Larvae that did not make a choice within ten minutes were not included in the statistical analysis. This explains the variable numbers of replicates for each test, ranging from 27 to 34. All behavioral assays were carried out within a 4 h time interval during larvae photophase.

### Statistics

Single sensillum recording data were analyzed using a Kruskal–Wallis test followed by nonparametric multiple comparisons using ‘nparcomp’ R package (type: Dunnett). For behavioral data, a Chi-squared test for given probabilities was used to verify the significance of caterpillars’ choice and a paired Student’s *t*-test was used to compare the time spent by larvae in each arm of the Y-tube olfactometer.

## Results

### Virtual screening of SlitOR24 and SlitOR25

#### Model performance

Each SlitOR model was parameterized with a LOO strategy, re-trained on the full training set once the best parameters were identified, and validated using the independent test set (Table 1). For SlitOR24, due to the limited number of active molecules in the training set, the model appeared to be mostly tuned to classify correctly the non-agonists. For SlitOR25, the model came with the benefit of an expanded applicability domain. However, the decrease in performance on the test set, compared to a previous preliminary model we conducted on this OR [20], is probably linked to the increased chemical diversity and thus to the complexity of the problem. Overall, both current SlitOR24 and SlitOR25 models had satisfying predictive abilities with MCC ≥ 0.4, and AUROC (area under the receiver operating characteristics curve) ≥ 0.8, and were suitable to prioritize compounds for experimental testing. To estimate the generalization error, five similar models were generated using alternative splits for preparing the training and test sets (Online Resources 4 and 5). When changing the distribution of compounds in the training and test sets, the overall performance of the predictive models remained similar compared to the final model. In details, the MCC was still above 0.4 and the AUROC ranged from 0.7 to 0.9 for both SlitOR24 and 25 models except for two alternate SlitOR24 models. For these two, no true positive compound was identified, mostly due to the small size of the dataset. One has to note that the false positive rate (i.e. the number of false positive prediction over the total number of inactive compounds) was higher for SlitOR25 models (0.17–0.50) than for SlitOR24 ones (0.00–0.10) and may lead to incorrectly classify non agonists and overestimate the number of compounds to be experimentally tested.

The current SlitOR25 and SlitOR24 machine learning models were used to virtually screen an in-house library of 158 natural volatile organic compounds. 28 and 67 molecules were predicted as agonists and within the applicability domain of SlitOR24 and SlitOR25 models, respectively, with 27 molecules in common (Online Resource 3). The 67 molecules predicted as agonists for SlitOR25 were re-screened by our previously published model [20] and 20 of them were predicted as agonists by both SlitOR25 models (Online Resource 3).

### Electrophysiological responses of SlitOR24 and SlitOR25 to the predicted agonists and non-agonists

**Fig. 1:**
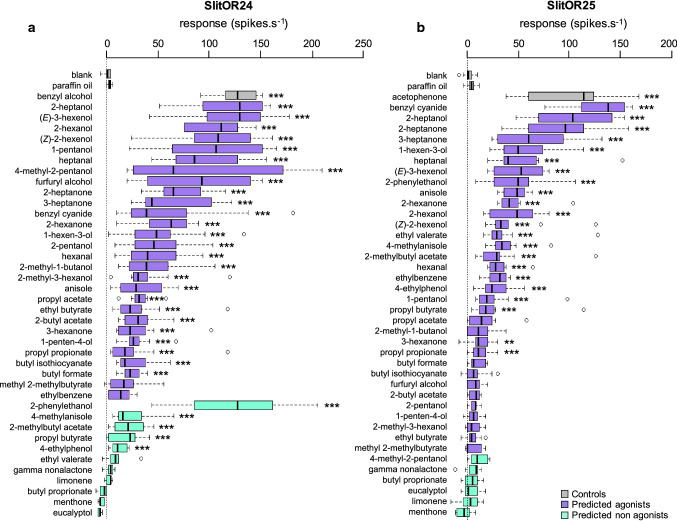
Responses of SlitOR24 and SlitOR25 to predicted ligands. Single-sensillum recording (SSR) responses (spikes.s^−1^) of *Drosophila* ab3A neurons expressing SlitOR24 (**a**) and SlitOR25 (**b**) during stimulation with QSAR model-predicted ligands. Gray bars represent negative controls (solvent and filter paper without odorant) and positive controls (known ligands for the respective OR [21]). Purple bars represent predicted agonists. Turquoise bars represent predicted non-agonists. All molecules were tested at a 10^–2^ dilution in paraffin oil. Box plots show the median (line), 25–75% percentiles (box), 10–90% percentiles (whisker), and outliers (dots). Asterisks indicate statistically significant differences between responses to the odorant and to the solvent alone (Kruskal–Wallis non parametric ANOVA followed by a Dunnett’s multiple comparison test, **p < 0.01, ***p < 0.001, *n* = 8–10 for predicted agonists, *n* = 5 for non-agonists)

To validate in silico predictions, we performed single sensillum recordings on *Drosophila* OSNs expressing SlitOR24 or SlitOR25 with a stimulus panel containing the 27 molecules predicted as agonists for both SlitOR24 and SlitOR25, 6 molecules predicted as agonists only for SlitOR25 by both the current and the published SlitOR25 models, and one molecule predicted as an agonist only for SlitOR24. We also tested five molecules predicted as non-agonists for both receptors (Online Resource 3) and one already known agonist for each OR as control [21]. In total, we tested 39 molecules on both receptors (28 + 11 and 33 + 6 predicted agonists + non-agonists for SlitOR24 and SlitOR25, respectively). As expected, both ORs responded to their respective positive control (Fig. 1).

For SlitOR24, 26 predicted agonists out of 28 were active (Fig. 1a), representing a 93% success rate of prediction. Among the six agonists predicted only for SlitOR25, four were active on SlitOR24 although they were not predicted as agonists by the model. Six molecules from the panel triggered responses above 100 spikes.s^−1^ (1-pentanol, (*Z*)-2-hexenol, 2-hexanol, (*E*)-3-hexenol, 2-heptanol and 2-phenylethanol, the latter eliciting the highest response), thus being as active as the previously identified agonist benzyl alcohol. Eight agonists triggered responses between 50 and 100 spikes.s^−1^ (1-hexen-3-ol, 2-hexanone, benzyl cyanide, 3-heptanone, 2-heptanone, furfuryl alcohol, 4-methyl-2-pentanol and heptanal).

For SlitOR25, 22 out of the selected 33 predicted agonists were active, representing a 67% success rate (Fig. 1b). As expected, the agonist predicted only for SlitOR24 did not elicit any SlitOR25 response. Two molecules from the panel triggered responses above 100 spikes.s^−1^ (2-heptanol and benzyl cyanide) and were more active than the previously identified agonist acetophenone, and six triggered responses between 50 and 100 spikes.s^−1^ (2-phenylethanol, (*E*)-3-hexenol, heptanal, 1-hexen-3-ol, 3-heptanone, 2-heptanone). None of the six non-agonists predicted for SlitOR25 elicited a significant response. In short, with a recall of 0.84 vs 1.00, both models were highly sensitive, even if the SlitOR25 model was less precise (0.93 vs 0.67) and specific (0.75 vs 0.35) than the SlitOR24 one, as expected by the evaluation metrics of the trained models (Online Resource 6).

### Behavioral effect of newly identified agonists

**Fig. 2:**
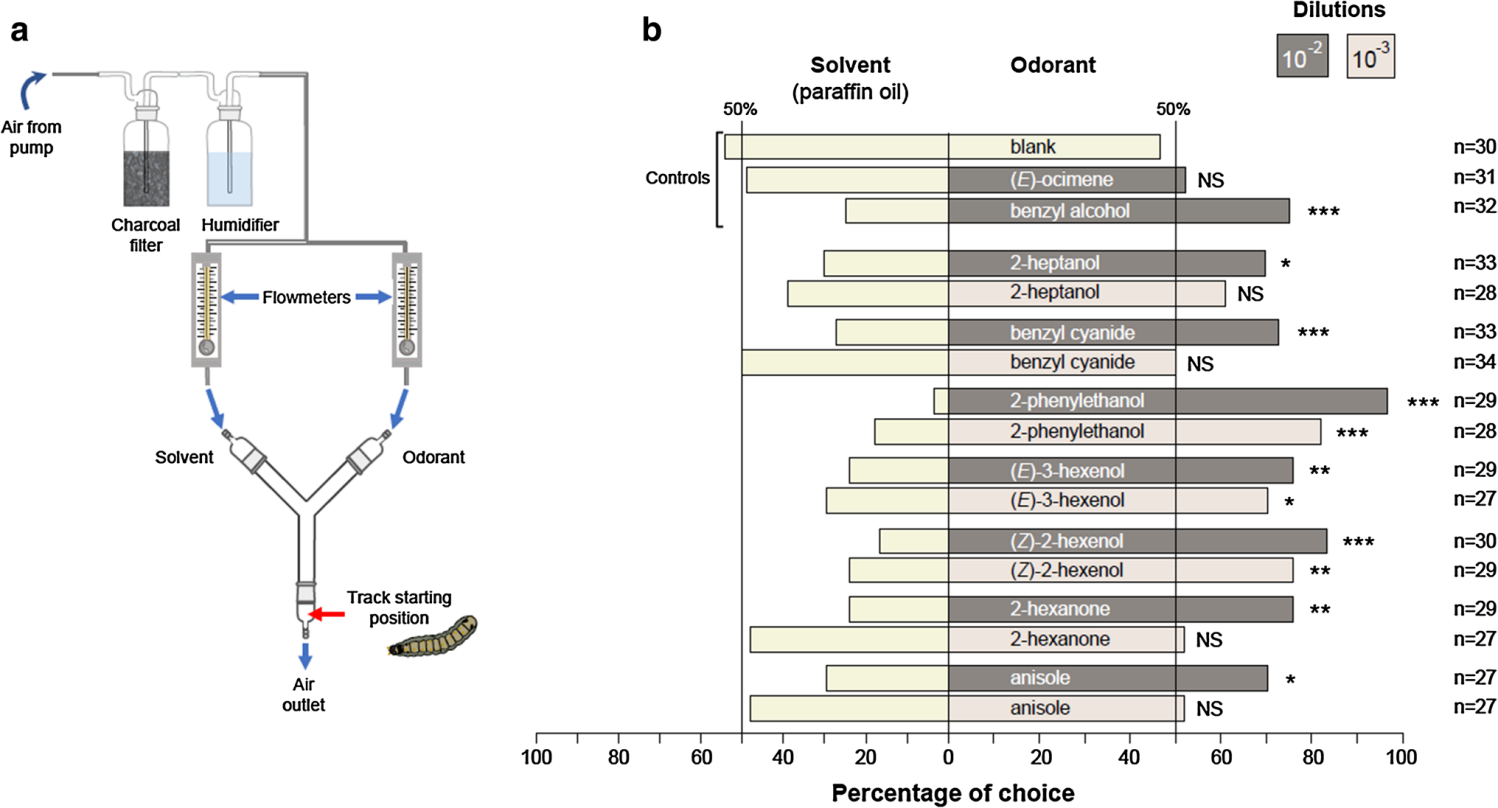
Behavioral responses (percentage of choice) of *S. littoralis* larvae to predicted ligands shown to be active on SlitOR24 and SlitOR25. **a** Experimental setup used to study caterpillar’s behavior. In this device, there is an air inlet, which circulates through two filters (active carbon and water bubbles), from where it passes to two flowmeters, to finally reach the Y-tube olfactometer. At the base of the olfactometer, the air outlet and the starting point for the larva are indicated. **b** Percentage of larval choice to (left/right): blank/blank (paraffin oil), neutral control (paraffin oil/ocimene), positive control (paraffin oil/benzyl alcohol), active ligands on ORs (paraffin oil/compounds). Dark gray bars at right represent the caterpillar’s choice at 10^–2^ dilution, and light gray bars represent caterpillar’s choice at 10^–3^ dilution. Asterisks indicate statistically significant preferences of larvae for the odorant side (Chi-squared test for given probabilities, *p < 0.05, **p < 0.01, ***p < 0.001, *NS* not significant). Numbers of replicates (*n*) are indicated on the right

**Fig. 3:**
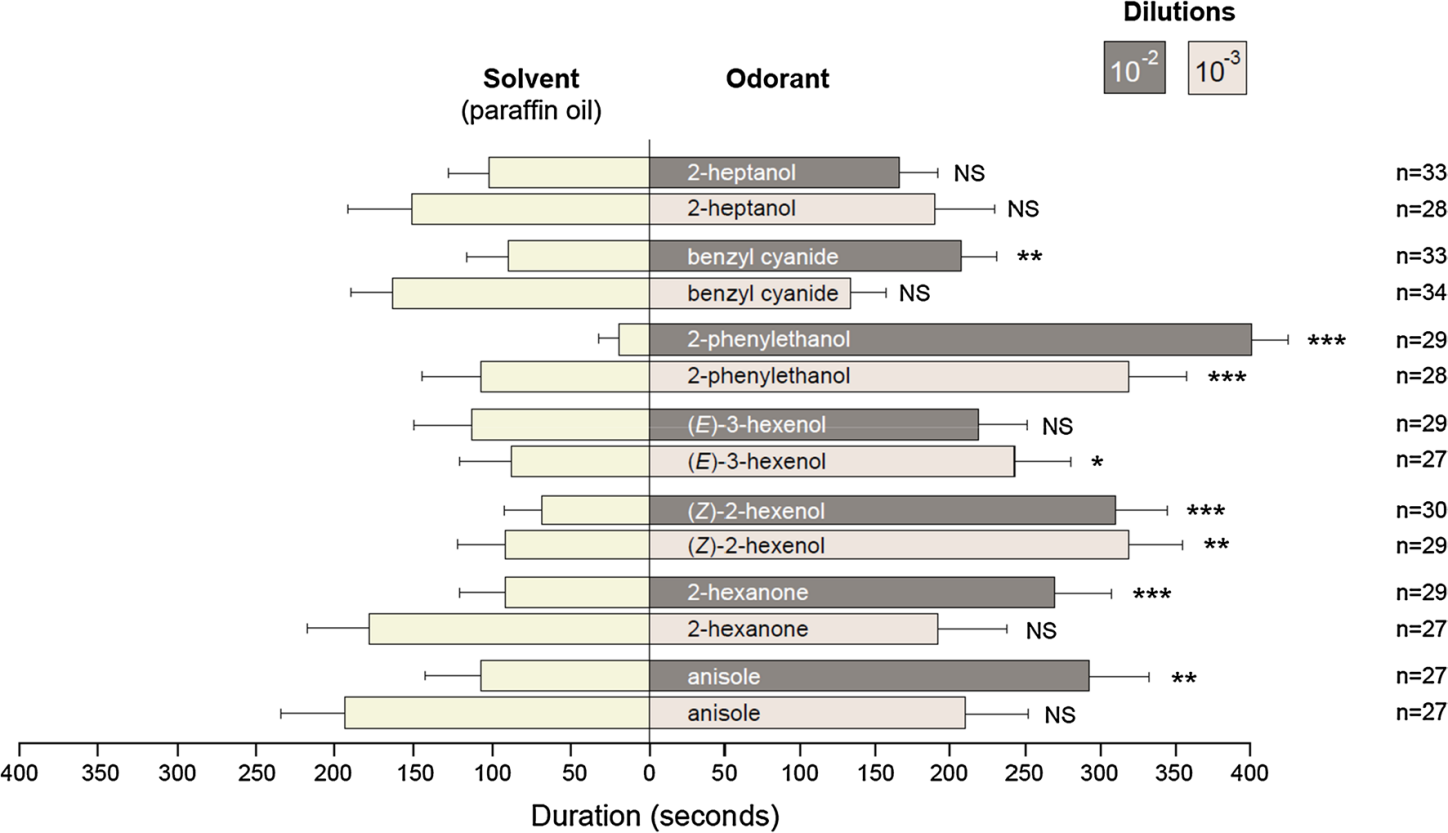
Behavioral responses (time in each arm) of *S. littoralis* larvae to predicted ligands shown to be active on SlitOR24 and SlitOR25. Time (in seconds) spent by the larvae in each arm on the Y-tube olfactometer. Bars at left represent the time spent in the arm containing the solvent (paraffin oil). Bars at right: Dark gray bars represent the time spent in the arm containing the odorant at 10^–2^ dilution, and light gray bars represent the time spent in the arm containing the odorant at 10^–3^ dilution. Asterisks indicate statistically significant differences between the time spent by larvae in each arm (Paired Student’s *t*-test, *p < 0.05, **p < 0.01, ***p < 0.001, *NS* not significant). Numbers of replicates are indicated on the right and error bars indicate SEM

The newly identified OR agonists were then tested for their effect on larvae behavior. In all behavioral experiments, larvae were starved for 16–20 h since previous experiments have shown that starved larvae are more motivated to orientate toward odor sources than satiated larvae and that such starvation has no impact in larval survival or mobility [28]. Before testing the effect of SlitOR24 and SlitOR25 ligands on the larval behavior, the experimental setup was first validated using different controls: paraffin oil (solvent), benzyl alcohol (known attractant) and (*E*)-ocimene (neutral) at dilution 10^–2^ [22]. As expected, larvae did not make any choice when exposed to both arms loaded with solvent. Larvae were statistically more attracted to the arm containing benzyl alcohol than to the control arm whereas no choice was observed using (*E*)-ocimene (Fig. 2). Then, seven of the molecules that elicited the highest neuronal responses in flies expressing SlitOR24 and SlitOR25 [(*Z*)-2-hexenol, (*E*)-3-hexenol, 2-phenylethanol, benzyl cyanide, 2-heptanol, anisole and 2-hexanone] were used in the same behavioral assay at two different dilutions (10^–2^ and 10^–3^). Results showed that for the 10^–2^ dilution, all molecules tested were attractive to the larvae (Fig. 2), with percentages of choice between 69.6% (2-heptanol) and 96.5% (2-phenylethanol). At the 10^–3^ dilution, larvae retained preference for three compounds: (*Z*)-2-hexenol, (*E*)-3-hexenol and 2-phenylethanol. Regarding the time spent in each arm (Fig. 3), larvae spent significantly more time in the arm containing five out of the seven molecules when tested at the 10^–2^ dilution: benzyl cyanide, (*Z*)-2-hexenol, 2-phenylethanol, anisole and 2-hexanone. At the 10^–3^ dilution, larvae spent more time in the arm containing three molecules: (*E*)-3-hexenol, 2-phenylethanol and 2-hexanone. Strikingly, the time spent by larvae on the arm containing (*E*)-3-hexenol was higher at the lowest dilution. In order correlate SSR responses and behavioural preferences, we constructed 2-D scatter plots for both SlitOR24 and 25, but r2 values were too low due to the low number of molecules tested in behavioral experiments to confidently evidence a positive correlation.

## Discussion

Reverse chemical ecology has recently appeared as a promising approach to identify behaviorally active semiochemicals that could be used for pest control strategies. In *Helicoverpa armigera* caterpillars, a combination of transcriptomic analyses, functional characterization of ORs and behavioral assays led to the identification of OR ligands that are behaviorally active (attractive and repulsive) for first-instar larvae [29]. A link between the activation of some ORs and attraction was also demonstrated in another species of pest caterpillars, the cotton leafworm *S. littoralis* [22]. These works thus showed that caterpillar ORs have a great potential as targets in reverse chemical ecology, yet the chances to identify behaviorally active molecules remain limited by the number of molecules tested on the target ORs. The incorporation of in silico modeling to the functional studies could fill this gap, since it has proven efficient when applied to the identification of new mosquito repellents [5, 18, 19]. Recently, we have published a proof-of-concept that revealed that such an approach can be extended to crop pest ORs [20]. Focusing on a single *S. littoralis* OR, SlitOR25, we could predict new agonists via machine learning that were indeed active on this OR, with a reasonable success rate of 28%. However, we did not investigate their behavioral activity. Anyhow, the chemical structures of the newly identified SlitOR25 agonists precluded their use for pest control, as most agonists were fluorinated compounds that cannot be used in the field [20].

In the present work, the objective was threefold. The first one was to improve our machine learning model for the prediction of agonists. The second objective was to predict natural, plant derivate, non-toxic and affordable agonists that would be compatible with pest control. The last objective was to investigate the behavioral activity of predicted agonists. To reach these objectives, we focused on the broadly tuned receptors SlitOR24 and SlitOR25 [21], whose activation has been linked to larvae attraction [22] and that were thus highly relevant for a reverse chemical ecology strategy. More, SlitOR25 has been used to establish the machine learning proof-of-concept on Lepidoptera ORs [20], and the data acquired (additional ligands) are perfectly suited to be used for model improvement.

First, we revealed that the QSAR models are highly precise since 67% and 93% of predicted agonists triggered a response in *Drosophila* olfactory neurons expressing SlitOR25 and SlitOR24, respectively. Even if the models lack specificity, notably for SlitOR25, they were sufficiently accurate to predict many new agonists. The SlitOR24 success rate was notably higher than what has been reported previously for Diptera. In *Drosophila*, more than 240,000 compounds were first screened in silico to find new OR ligands [15]. OR-optimized descriptors allowed to rank the untested molecules, identifying the top 500 hits for each OR. Predicted compounds were experimentally tested on nine ORs, showing 71% of success rate compared to only 10% when using non-predicted odors [15]. In mosquitoes, Tauxe et al. 2013 obtained a ~ 30% success rate when trying to identify CO_2_ receptor activators by using molecular descriptors [18]. In a second round of in silico prediction, they increased prediction accuracy through a SVM-based approach, yielding an improved success rate of 74%. In the present work, while the SlitOR24 model appeared exceptionally precise to identify true agonists (93%), it has to be noticed that it did miss some of them. Some of the molecules predicted as agonists only for SlitOR25 appeared to be agonists for SlitOR24 (false negative rate of 18%). Reversely, the SlitOR25 model was less efficient to identify true agonists (precision of 67% and a false positive rate of 65%), but was highly sensitive and succeeded in predicting all the non-agonists. More, combining the SlitOR25 model with the previously published one [20] (Online Resource 3) guided us to prioritize the most promising compounds. As already reported in mosquitoes [18], such results suggest that model combination, in addition to cumulative experimental data to feed models, offer a way to improve insect OR ligand identification.

One has to keep in mind that our models are based on experimental data obtained from ORs expressed in the empty neuron system of *Drosophila*, which lacks perireceptor proteins such as odorant-binding proteins and odorant-degrading enzymes [12]. We cannot rule out that response spectra of caterpillar ORs expressed in a fly neuron may somehow differ from the response of the corresponding caterpillar neurons, leading to a potential confounding effect on the modeling. However, we have previously shown that, when expressed in the empty neuron system, SlitOR24 and SlitOR25 exhibit exactly the same response spectrum than the two corresponding olfactory neurons from *S. littoralis* adult antennae (see Supplementary Figure S3 in [21]). Thus, we can be confident in the use of models based on empty neuron SSR data for identifying molecules active on *S. littoralis* caterpillars.

To reach the second objective, the QSARs have been used here to screen an in-house virtual library of plant compounds, while our previous efforts focused on a large subset of the Pubchem database selected on physico-chemical properties that led to the identification of structurally related, mainly fluorinated, predicted ligands [20]. Through this approach, we have identified new agonists for SlitOR24 and SlitOR25 (more or equally active as previously identified ligands), greatly extending their initially described response spectra [21]. Both ORs presented a large overlapping receptive range, including aliphatic alcohols, aromatic compounds and green leaf volatiles. Interestingly, a large majority (74%) of predicted ligands for SlitOR25 were also active on SlitOR24. This suggests that the binding pocket of both ORs would be quite similar and opens up further studies on structure–function relationships. The tridimensional structure of an insect (*Machilis hrabei*) OR has been recently elucidated [30] and even if the sequence identity between MhraOR5 and SlitOR24 or 25 is low (< 20%), one can try to extrapolate the corresponding binding pockets. Interestingly, in line with the experimental data, a multiple sequence alignment suggests that the residues from the putative odorant-binding sites of SlitOR24 and 25 are highly conserved (Online Resource 7).

The behavioral effects of the new ligands that elicited high neuronal responses were investigated on larvae, and all proved to be attractive. These data not only confirmed the former hypothesis that SlitOR24 and OR25 activation is linked to larval attraction [22], but also demonstrated that reverse chemical ecology is efficient in predicting behaviorally active odorants. Interestingly, many of these new attractants for *S. littoralis* larvae have never been reported to be relevant cues for adults or larvae on this species. Among the new ligands for SlitOR25, benzyl cyanide (a nitrogenous aromatic compound) induced the highest OSN firing rate and a high attraction rate. It has been shown previously that benzyl cyanide is a herbivore-induced volatile emitted by diverse plants, like the black poplar *Populus nigra* and Brussels sprouts *Brassicae oleracea* [31, 32]. On the one hand, such signal indicates actual presence of herbivores, and thus the possible presence of adequate food for larvae. On the other hand, benzyl cyanide has also been reported to be attractive to different parasitoid species that use this cue to detect the presence of host larvae [31, 32]. Benzyl cyanide is also naturally emitted by some insect species, and is notably known as a male anti-aphrodisiac pheromone in the desert locust [33] as well as in the butterfly *Pieris brassicae.* In this latter species, it is transferred to the females while mating, making them less attractive to conspecific males [34]. In turn, this anti-aphrodisiac is exploited by parasitoid wasps such as *Trichogramma brassicae* to detect laid eggs for further parasitization [35]. The most potent attractant for *S. littoralis* larvae at both doses tested was 2-phenylethanol, an aromatic compound that induced the highest firing rate in OSNs expressing SlitOR24. 2-phenylethanol is released by flowers, fruits or vegetative tissues of a large array of plants from a multitude of families [36] and it may be important for caterpillar foraging behavior. It is documented as one of the most attractive compounds—together with phenylacetaldehyde—for *H. armigera* adults [37, 38] and elicited high neuronal responses in *Heliothis virescens* females [39].

Although we propose here a probable role in caterpillar foraging behavior, the potential ecological significance of these *S. littoralis* larval attractants remains to be determined, as well as their behavioral effects on adults, in which SlitOR24 and 25 are also expressed in antennae [40, 41]. Anyhow, our work shows that reverse chemical ecology can be applied efficiently to identify behaviorally-active volatiles that could ultimately implement semiochemical-based control strategies against agricultural pests. Improved membrane protein tridimensional structure resolution [30, 42] and prediction [43, 44] will give access to structural details of the odorant-binding pocket of insect ORs then contributing to expand the chemical space to be explored by structure-based virtual screening.

### Supplementary Information

Below is the link to the electronic supplementary material.

Supplementary file1 (PDF 1020 KB)

Supplementary file2 (PDF 962 KB)

Supplementary file3 (PDF 1117 KB)

Supplementary file4 (DOCX 27 KB)

Supplementary file5 (DOCX 16 KB)

Supplementary file6 (DOCX 15 KB)

Supplementary file7 (DOCX 16 KB)

## Data Availability

All in silico data and weka model files have been deposited on GitHub (https://github.com/chemosim-lab/SlitOR_data). Transformant flies are available on request to Emmanuelle Jacquin-Joly (emmanuelle.joly@inrae.fr) or Nicolas Montagné (nicolas.montagne@sorbonne-universite.fr).

## Abbreviations

AUROC: Area Under the Receiver-Operating Characteristics curve
kNN: K-nearest neighbors
LOO: Leave-one-out
MCC: Matthews correlation coefficient
OR: Odorant receptor
OSN: Olfactory sensory neuron
QSAR: Quantitative-structure–activity relationship
SSR: Single sensillum recordings
SVC: Support Vector Classifier
SVM: Support Vector Machine

## Code availability

All the binary files necessary to screen the database with the machine-learning models, the code used to define the applicability domain of the models, a description of the content of each file and how to use them are made available on GitHub (https://github.com/chemosim-lab/SlitOR_data).
