# Supplementary material for: Reverse chemical ecology in a moth: machine learning on odorant receptors identifies new behaviorally active agonists
Source: Cell Mol Life Sci. 2021 Aug 27;78(19-20):6593–603. doi: 10.1007/s00018-021-03919-2 (PMC8558168; doi:10.1007/s00018-021-03919-2)
Supplement: Supplementary file 1 — Supplementary file1 (PDF 1020 KB) [file 18_2021_3919_MOESM1_ESM.pdf]

Reverse chemical ecology in a moth: machine learning on odorant receptors identifies new behaviorally active agonists

CMLS

Gabriela Caballero-Vidal[1§¤], Cédric Bouysset[2§], Jérémy Gévar[1], Hayat Mbouzid[1], Céline Nara[1], Julie Delaroche[1], Jérôme Golebiowski[2,3], Nicolas Montagné[1*], Sébastien Fiorucci[2*], & Emmanuelle Jacquin-Joly[1*]

[1] INRAE, Sorbonne Université, CNRS, IRD, UPEC, Université de Paris, Institute of Ecology and Environmental Sciences of Paris, Versailles 78000, France
[2] Université Côte d'Azur, CNRS, Institut de Chimie de Nice UMR7272, Nice 06000, France
[3] Department of Brain and Cognitive Sciences, Daegu Gyeongbuk Institute of Science and Technology, Daegu 711-873, South Korea
[¤] present address: Disease Vector Group, Chemical Ecology, Department of Plant Protection Biology, Swedish University of Agricultural Sciences, Alnarp, Sweden
Max Planck Centre Next Generation Chemical Ecology, Uppsala, Sweden

[§]both authors contributed equally to the work

*Corresponding authors:
Emmanuelle Jacquin-Joly
emmanuelle.joly@inrae.fr

Sébastien Fiorucci
sebastien.fiorucci@univ-cotedazur.fr

Nicolas Montagné
nicolas.montagne@sorbonne-universite.fr

Online Resource 1.
In-house library of plant volatile organic compounds

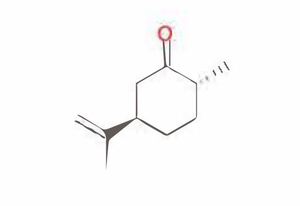<br>(+)-dihydrocarvone<br>5524-05-0	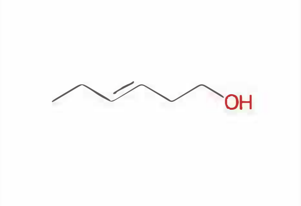<br>(E)-3-hexen-1-ol<br>928-97-2	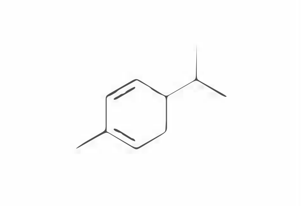<br>(R)-α-phellandrene<br>4221-98-1	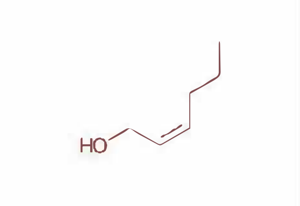<br>(Z)-2-hexen-1-ol<br>928-94-9	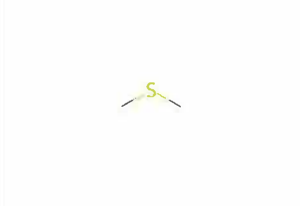<br>(methylthio)methane<br>75-18-3
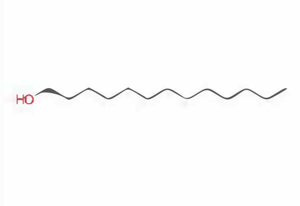<br>1- tridecanol<br>112-70-9	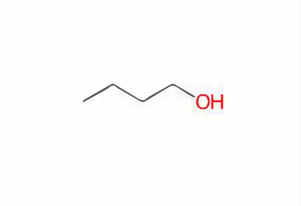<br>1-butanol<br>71-36-3	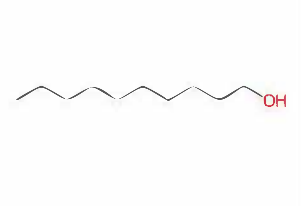<br>1-decanol<br>112-30-1	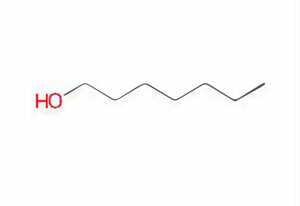<br>1-heptanol<br>111-70-6	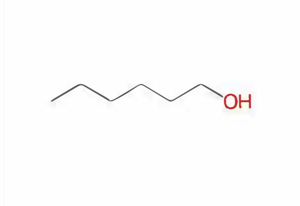<br>1-hexanol<br>111-27-3
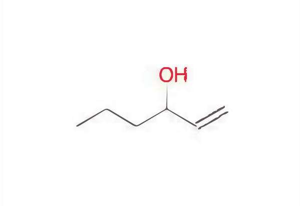<br>1-hexen-3-ol<br>4798-44-1	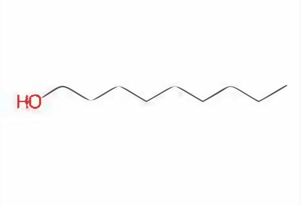<br>1-nonanol<br>143-08-8	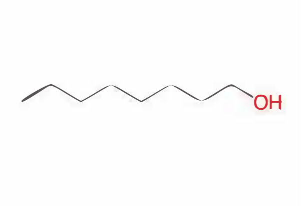<br>1-octanol<br>111-87-5	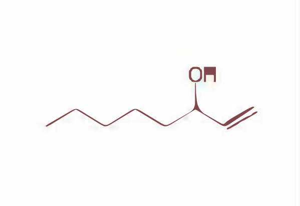<br>1-octen-3-ol	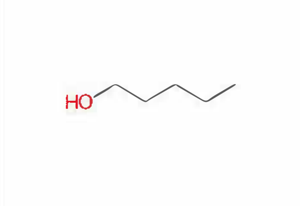<br>1-pentanol<br>71-41-0
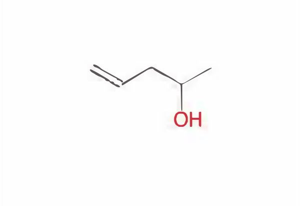<br>1-penten-4-ol<br>625-31-0	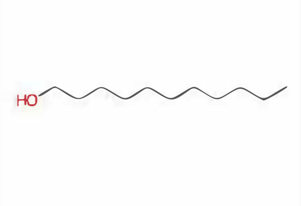<br>1-undecanol<br>112-42-5	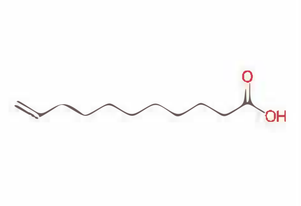<br>1-undecenoic acid<br>112-38-9	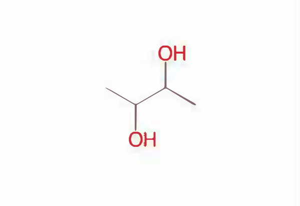<br>2,3-butanediol<br>513-85-9	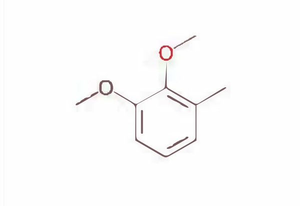<br>2,3-dimethoxytoluene<br>4463-33-6
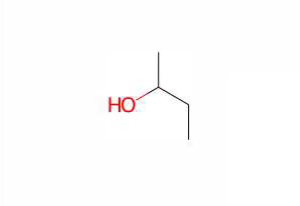<br>2-butanol<br>78-92-2	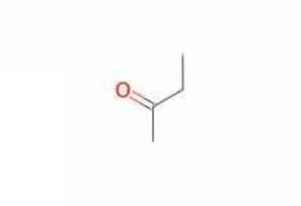<br>2-butanone<br>78-93-3	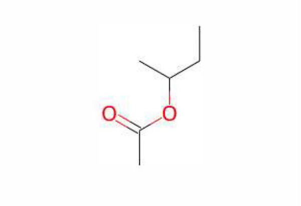<br>2-butyl acetate<br>105-45-4	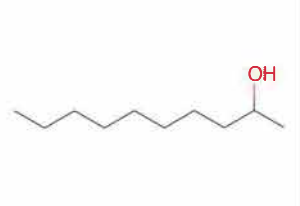<br>2-decanol<br>1120-06-5	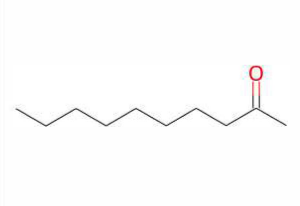<br>2-decanone<br>693-54-9
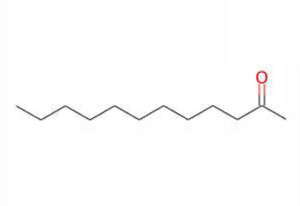<br>2-dodecanone<br>6175-49-1	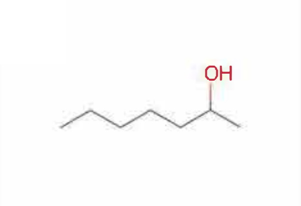<br>2-heptanol<br>543-49-7	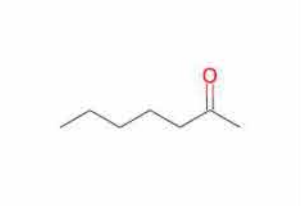<br>2-heptanone<br>110-43-0	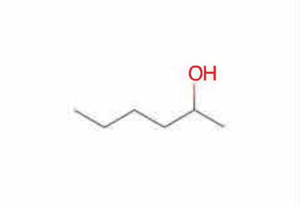<br>2-hexanol<br>626-93-7	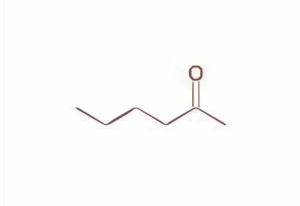<br>2-hexanone<br>591-78-6
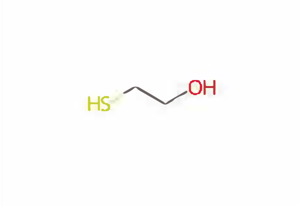<br>2-mercaptoethanol	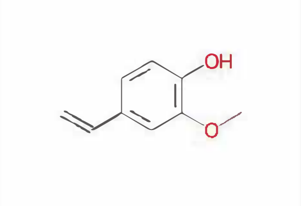<br>2-methoxy-4-vinylphenol<br>7786-61-0	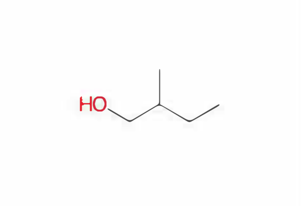<br>2-methyl-1-butanol<br>137-32-6	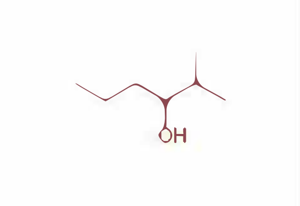<br>2-methyl-3-hexanol<br>617-29-8	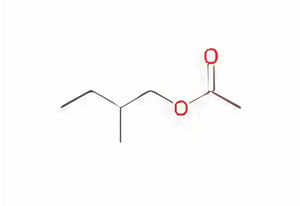<br>2-methylbutyl acetate<br>624-41-9
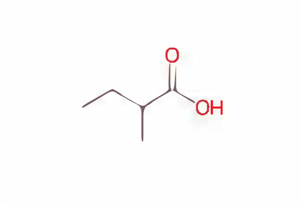<br>2-methylbutyric acid<br>116-53-0	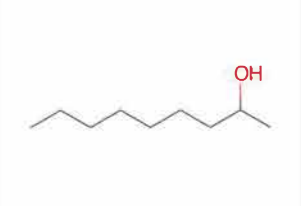<br>2-nonanol<br>628-99-9	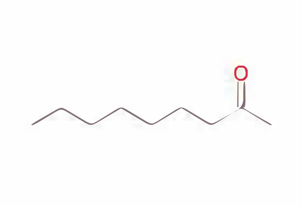<br>2-nonanone<br>821-55-6	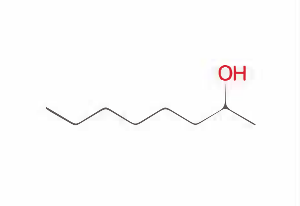<br>2-octanol	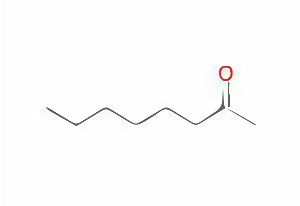<br>2-octanone<br>111-13-7

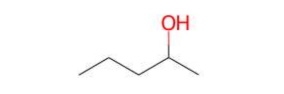<br>2-pentanol<br>6032-29-7	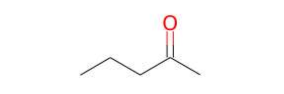<br>2-pentanone<br>107-87-9	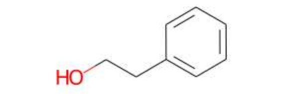<br>2-phenyl ethanol<br>60-12-8	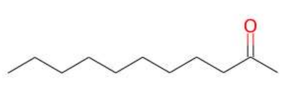<br>2-undecanone<br>112-12-9	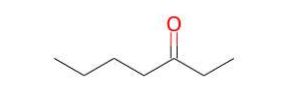<br>3-heptanone<br>106-35-4
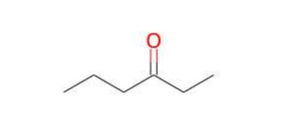<br>3-hexanone<br>589-38-8	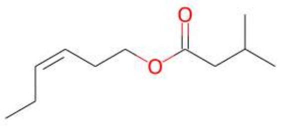<br>3-hexenyl 3-methylbutanoate<br>35154-45-1	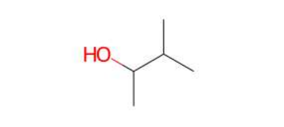<br>3-methyl-2-butanol<br>598-75-4	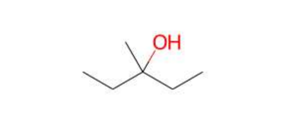<br>3-methyl-3-pentanol<br>77-74-7	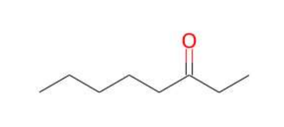<br>3-octanone<br>106-68-3
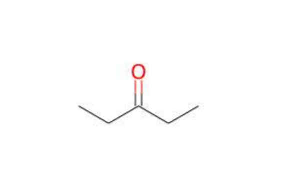<br>3-pentanone<br>96-22-0	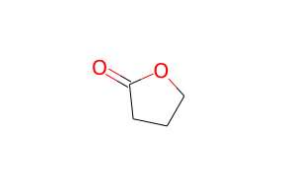<br>4-butyrolactone<br>96-48-0	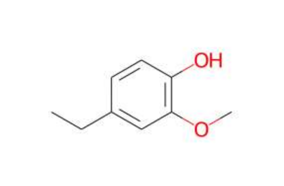<br>4-ethylguaiacol	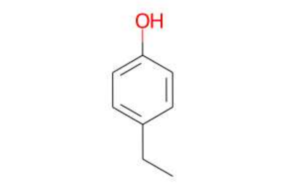<br>4-ethylphenol<br>123-07-9	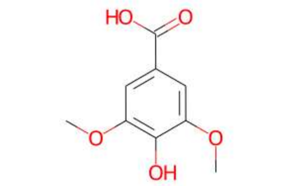<br>4-hydroxy 3,5 benzoic acid<br>530-57-4
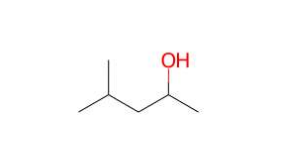<br>4-methyl-2-pentanol<br>108-11-2	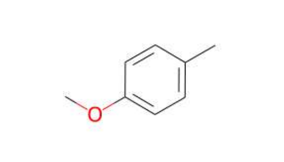<br>4-methylanisole<br>100-66-3	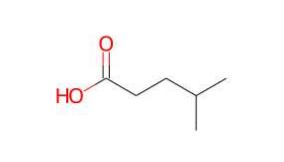<br>4-methylvaleric acid<br>646-07-1	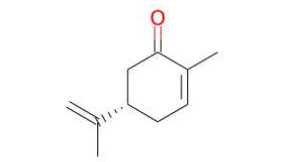<br>D-carvone<br>2244-16-8	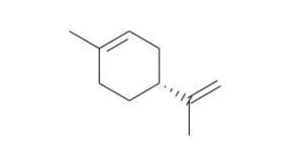<br>D-limonene<br>5989-27-5
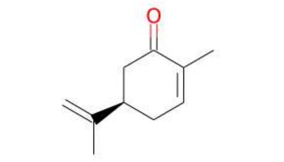<br>L-carvone<br>6485-40-1	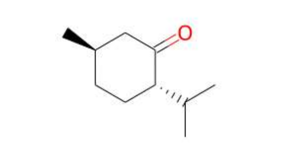<br>L-menthone<br>14073-97-3	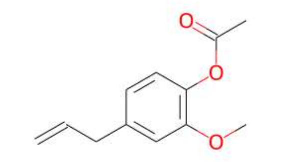<br>acetyleugenol	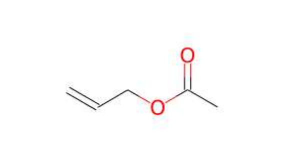<br>allyl acetate<br>591-87-7	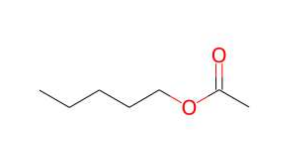<br>amyl acetate<br>628-63-7
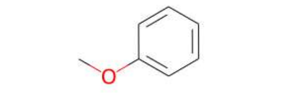<br>anisole<br>100-66-3	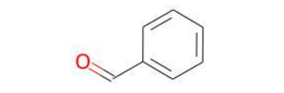<br>benzaldehyde<br>100-52-7	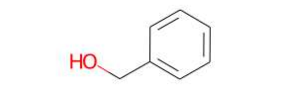<br>benzyl alcohol<br>100-51-6	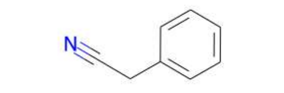<br>benzyl cyanide<br>140-29-4	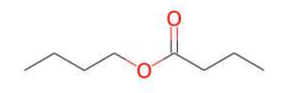<br>butyl butyrate<br>109-21-7
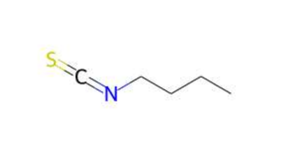<br>butyl isothiocyanate<br>592-82-5	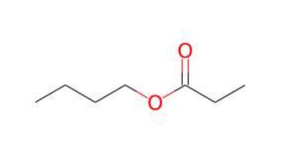<br>butyl propionate<br>590-01-2	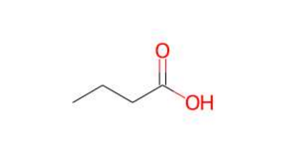<br>butyric acid<br>107-92-6	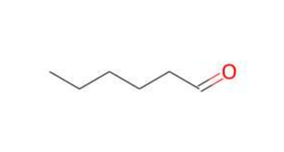<br>caproaldehyde (hexanal)<br>66-25-1	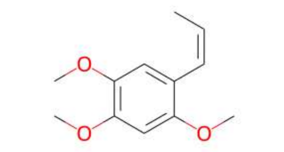<br>cis-2,4,5-trimethoxy-1-propenylbenzene
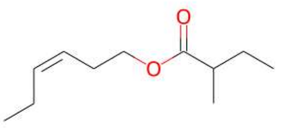<br>cis-3-hexenyl 2-methylbutanoate<br>53398-85-9	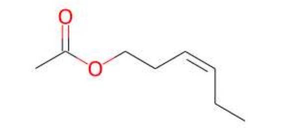<br>cis-3-hexenyl acetate<br>3681-71-8	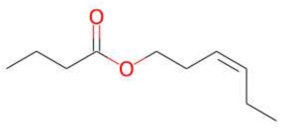<br>cis-3-hexenyl butyrate<br>16491-36-4	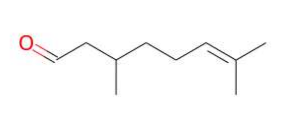<br>citronellal<br>106-23-0	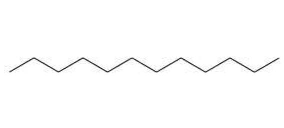<br>dodecane

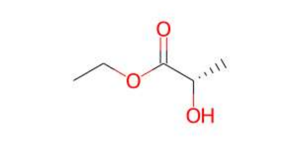
ethyl (S)-(-)-lactate
687-47-8

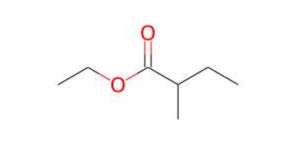
ethyl 2-methylbutyrate
7452-79-1

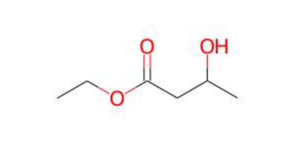
ethyl 3-hydroxybutyrate
5405-41-4

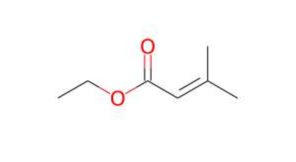
ethyl 3-methylcrotonate
638-10-8

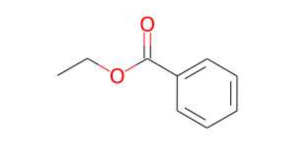
ethyl benzoate
93-89-0

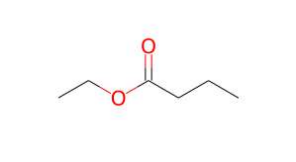
ethyl butyrate
105-54-4

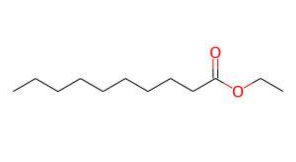
ethyl caprate
110-38-3

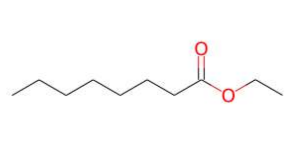
ethyl caprylate

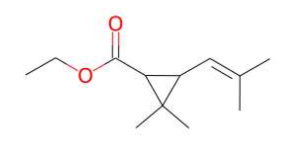
ethyl chrysanthemumate
97-41-6

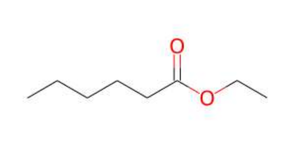
ethyl hexanoate

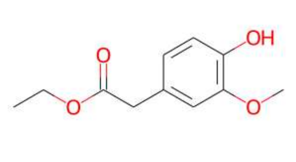
ethyl homovanillate

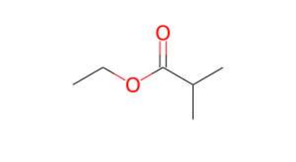
ethyl isobutyrate
97-62-1

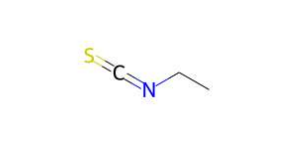
ethyl isothiocyanate
542-85-8

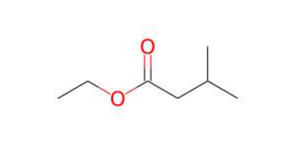
ethyl isovalerate
108-64-5

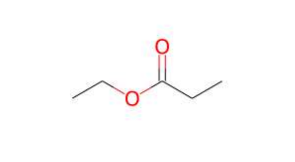
ethyl propionate
105-37-3

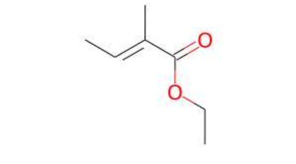
ethyl tiglate
5837-78-5

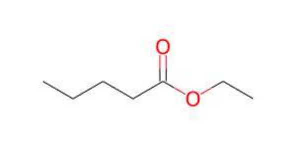
ethyl valerate
539-82-2

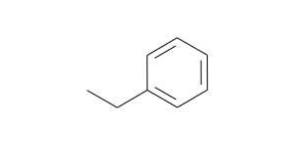
ethylbenzene
100-41-4

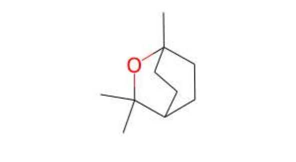
eucalyptol
470-82-6

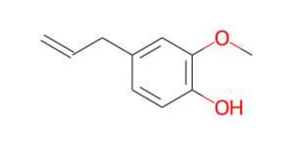
eugenol

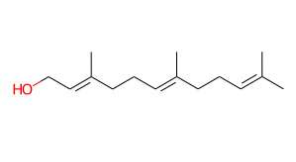
farnesol

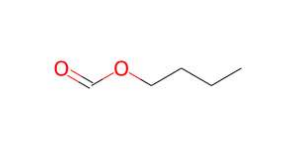
formic acid butyl ester
592-84-7

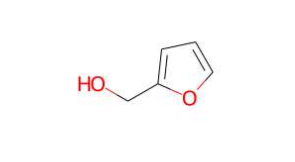
furfuryl alcohol
98-00-0

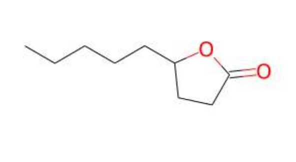
gamma-nonanoic lactone
104-61-0

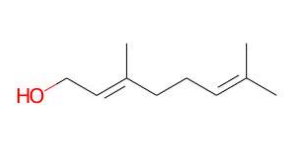
geraniol
106-24-1

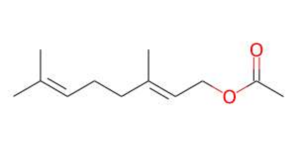
geranyl acetate
16409-44-2

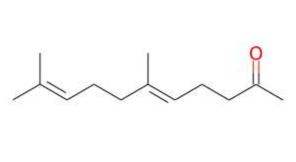
geranylacetone
3796-70-1

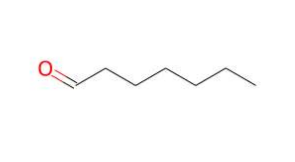
heptaldehyde
111-71-7

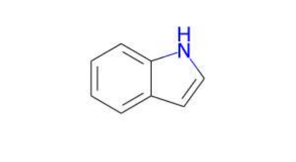
indole
120-72-9

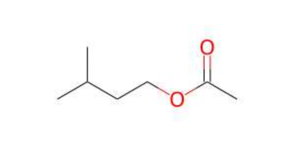
isoamyl acetate
123-92-2

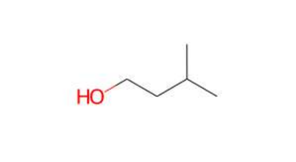
isoamyl alcohol

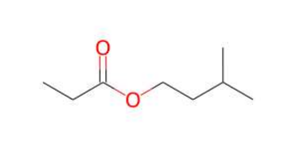
isoamyl propionate
105-68-0

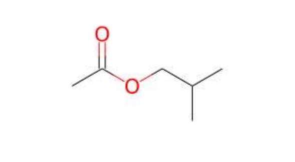
isobutyl acetate
110-19-0

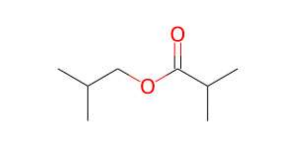
isobutyl isobutyrate
97-85-8

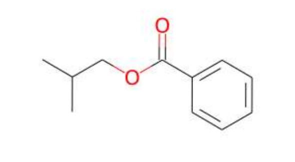
isobutylbenzoate

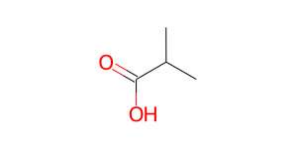
isobutyric acid
79-31-2

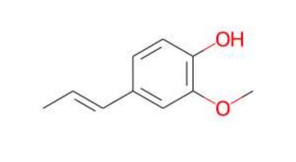
isoeugenol

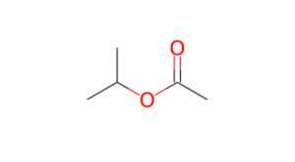
isopropyl acetate
108-21-4

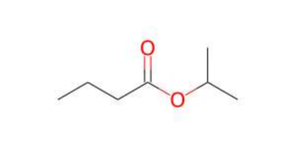
isopropyl butyrate
638-11-9

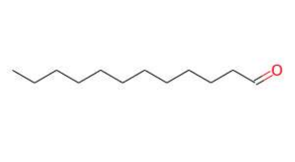
laurinaldehyde
112-54-9

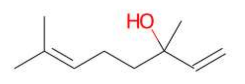

linalool
78-70-6

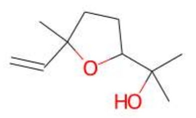

linalool oxide
60047-17-8

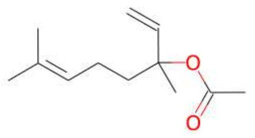

linalyl acetate
115-95-7

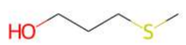

methionol
505-10-2

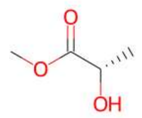

methyl (S)-(-)-lactate
27871-49-4

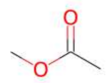

methyl acetate

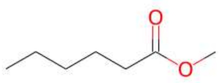

methyl caproate
106-70-7

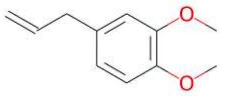

methyl eugenol

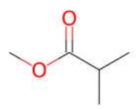

methyl isobutyrate
547-63-7

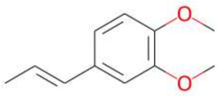

methyl isoeugenol

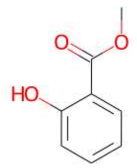

methyl salicylate

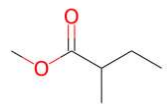

methyl-2-methylbutyrate
868-57-5

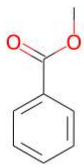

methylbenzoate

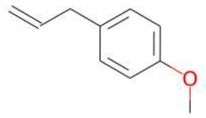

methylchavicol
140-67-0

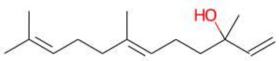

nerolidol

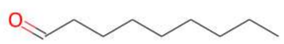

nonanal
124-19-6

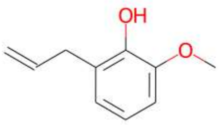

o-eugenol

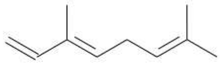

ocimene
13877-91-3

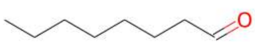

octanal

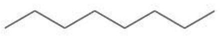

octane
111-65-9

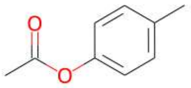

p-tolyl acetate
140-39-6

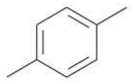

p-xylene

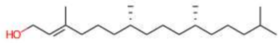

phytol

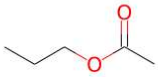

propyl acetate
109-60-4

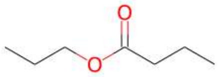

propyl butyrate
105-66-8

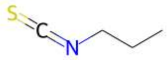

propyl isothiocyanate
628-30-8

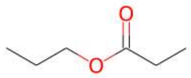

propyl propionate
106-36-5

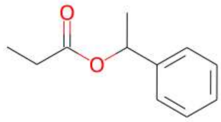

styrallyl propionate
120-45-6

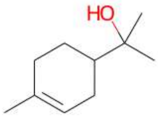

terpineol anhydre
8000-41-7

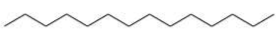

tetradecane
629-59-4

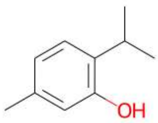

thymol

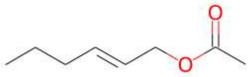

trans-2-hexenyl acetate
2497-18-9

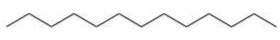

tridecane

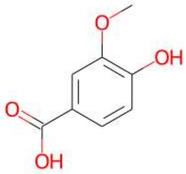

vanilic acid

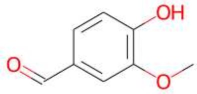

vanillin

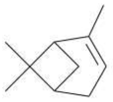

α-pinene
80-56-8

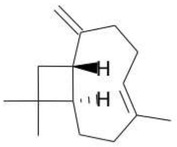

β-caryophyllene
87-44-5

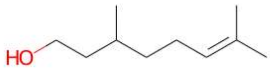

β-citronellol
106-22-9

Online Resource 1.
in-house library of plant volatile organic compounds
