# Supplementary material for: Reverse chemical ecology in a moth: machine learning on odorant receptors identifies new behaviorally active agonists
Source: Cell Mol Life Sci. 2021 Aug 27;78(19-20):6593–603. doi: 10.1007/s00018-021-03919-2 (PMC8558168; doi:10.1007/s00018-021-03919-2)
Supplement: Supplementary file 2 — Supplementary file2 (PDF 962 KB) [file 18_2021_3919_MOESM2_ESM.pdf]

Reverse chemical ecology in a moth: machine learning on odorant receptors identifies new behaviorally active agonists

CMLS

Gabriela Caballero-Vidal[1§¤], Cédric Bouysset[2§], Jérémy Gévar[1], Hayat Mbouzid[1], Céline Nara[1], Julie Delaroche[1], Jérôme Golebiowski[2,3], Nicolas Montagné[1*], Sébastien Fiorucci[2*], & Emmanuelle Jacquin-Joly[1*]

[1] INRAE, Sorbonne Université, CNRS, IRD, UPEC, Université de Paris, Institute of Ecology and Environmental Sciences of Paris, Versailles 78000, France
[2] Université Côte d'Azur, CNRS, Institut de Chimie de Nice UMR7272, Nice 06000, France
[3] Department of Brain and Cognitive Sciences, Daegu Gyeongbuk Institute of Science and Technology, Daegu 711-873, South Korea
[¤] present address: Disease Vector Group, Chemical Ecology, Department of Plant Protection Biology, Swedish University of Agricultural Sciences, Alnarp, Sweden
Max Planck Centre Next Generation Chemical Ecology, Uppsala, Sweden

[§]both authors contributed equally to the work

*Corresponding authors:
Emmanuelle Jacquin-Joly
emmanuelle.joly@inrae.fr

Sébastien Fiorucci
sebastien.fiorucci@univ-cotedazur.fr

Nicolas Montagné
nicolas.montagne@sorbonne-universite.fr

Online Resource 2

Name	CAS	Structure	Expected Activity on SlitOR24	Dataset SlitOR24	Expected Activity on SlitOR25	Dataset SlitOR25	SMILES
α-humulene	6753-98-6	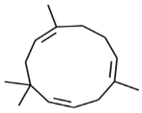	0	Test	0	Training	C/C1=C\CC/C(C)=C/CC© (C)/C=C/C1
β-caryophyllene	87-44-5	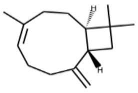	0	Training	0	Training	C=C1CC/C=C(\C)CC[C@@H]2[C@ @H]1CC2(C)C
3-carene	13466-78-9	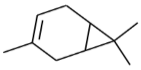	0	Training	0	Training	CC1=CCC2C(C1)C2(C)C
α-copaene	3856-25-5	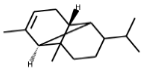	0	Training	0	Training	CC1=CC[C@@H]2C3C(C(C)C)CCC2 ©[C@@H]13
(E,E)-α-farnesene	502-61-4	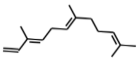	0	Training	0	Test	C=C/C(C)=C/C/C=C(\C)CCC=C(C)C
α-pinene	80-56-8	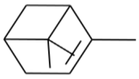	0	Training	0	Training	CC1=CCC2CC1C2(C)C
β-pinene	127-91-3	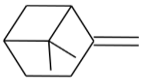	0	Test	0	Training	C=C1CCC2CC1C2(C)C
β-myrcene	123-35-3	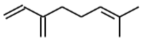	0	Training	0	Test	C=CC(=C)CCC=C(C)C
(E)-ocimene	13877-91-3	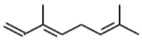	0	Training	0	Training	C=C/C(C)=C/CC=C(C)C
indole	120-72-9	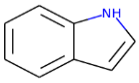	0	Training	0	Training	c1ccc2[nH]ccc2c1
DMNT	26049-69-4	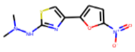	0	Training	0	Training	CN(C)Nc1nc(-c2ccc([N+](=O)[O-])o2)cs1
TMTT	62235-06-7	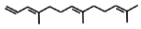	0	Training	0	Training	C=C/C=C(\C)CC/C=C(\C)CCC=C(C)C

(Z)-jasmone	488-10-8	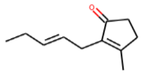	0	Test	0	Test	CCC=CCC1=C(C)CCC1=O
decanal	112-31-2		0	Training	0	Training	CCCCCCCCCC=O
nonanal	124-19-6		0	Training	0	Test	CCCCCCCCC=O
1-nonanol	143-08-8		0	Test	0	Training	CCCCCCCCCO
1-octanol	111-87-5		0	Training	0	Training	CCCCCCCCO
carvacrol	499-75-2	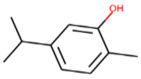	0	Training	0	Test	Cc1ccc(C(C)C)cc1O
estragole	140-67-0	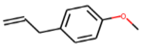	0	Training	0	Test	C=CCc1ccc(OC)cc1
eugenol	97-53-0	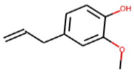	0	Training	0	Training	C=CCc1ccc(O)c(OC)c1
geraniol	106-24-1	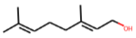	0	Training	0	Test	CC(C)=CCC/C(C)=C/CO
(±)-linalool	78-70-6	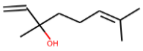	0	Training	0	Training	C=CC(C)(O)CCC=C(C)C
(±)-nerolidol	7212-44-4	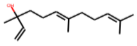	0	Training	0	Training	C=CC(C)(O)CC/C=C(\C)CCC=C(C)C
(±)-phytol	7541-49-3	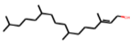	0	Training	0	Training	C/C(=C\CO)CCCC(C)CCCC(C)CCCC(C)C
thymol	89-83-8	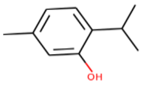	0	Training	0	Training	Cc1ccc(C(C)C)c(O)c1
(E,E)-farnesol	106-28-5	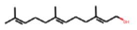	0	Test	0	Test	CC(C)=CCC/C(C)=C/CC/C(C)=C/CO

EDD	CHEMBL1271376	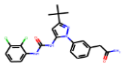	0	Training	0	Training	CC(C)(C)c1cc(NC(=O)Nc2cccc(Cl)c2Cl)n(-c2cccc(CC(N)=O)c2)n1
methyl jasmonate	1101843-02-0	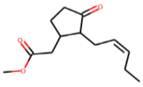	0	Test	0	Training	CC/C=C\CC1C(=O)CCC1CC(=O)OC
sulcatone	110-93-0	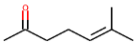	0	Test	0	Test	CC(=O)CCC=C(C)C
(Z,E)-9,11-14:OAc	50767-79-8	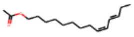	0	Training	0	Training	CC/C=C/C=C\CCCCCCCCOC(C)=O
(Z,E)-9,12-14:OAc	31654-77-0	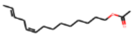	0	Training	0	Training	C/C=C/C/C=C\CCCCCCCCOC(C)=O
(Z)7-12:OAc	14959-86-5	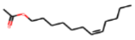	0	Training	0	Training	CCCC/C=C\CCCCCCOC(C)=O
(Z)9-14:OAc	16725-53-4	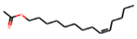	0	Training	0	Training	CCCC/C=C\CCCCCCCCOC(C)=O
(Z)9-14: OH	35153-15-2	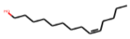	0	Training	0	Training	CCCC/C=C\CCCCCCCCO
14:OAc	638-59-5	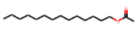	0	Test	0	Training	CCCCCCCCCCCCCCOC(C)=O
(E)11-14:OAc	33189-72-9	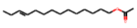	0	Training	0	Training	CC/C=C/CCCCCCCCCCOC(C)=O
(E)2-hexenal	6728-26-3	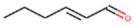	0	Test	1	Training	CCC/C=C/C=O
methyl salicylate	119-36-8	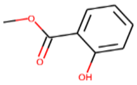	0	Training	1	Training	COC(=O)c1ccccc1O
methyl benzoate	93-58-3	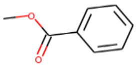	0	Test	1	Test	COC(=O)c1ccccc1
benzyl methyl ether	538-86-3	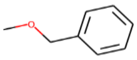	0	Training	1	Test	COCc1ccccc1

1-indanone	83-33-0	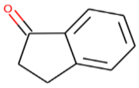	0	Training	1	Training	O=C1CCc2ccccc21
(Z)3-hexenyl acetate	3681-71-8	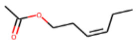	1	Test	0	Test	CC/C=C\CCOC(C)=O
(Z)11-14:OAc	20711-10-8	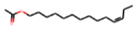	1	Training	0	Training	CC/C=C\CCCCCCCCCCOC(C)=O
benzaldehyde	100-52-7	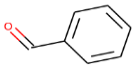	1	Test	1	Training	O=Cc1ccccc1
phenylacetaldehyde	122-78-1	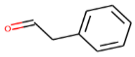	1	Training	1	Test	O=CCc1ccccc1
(E)2-hexenol	928-95-0	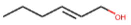	1	Training	1	Training	CCC/C=C/CO
(Z)3-hexenol	928-96-1	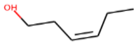	1	Training	1	Training	CC/C=C\CCO
1-hexanol	111-27-3	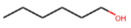	1	Training	1	Training	CCCCCCO
1-heptanol	111-70-6		1	Training	1	Test	CCCCCCCO
benzyl alcohol	100-51-6	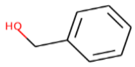	1	Training	1	Test	OCc1ccccc1
acetophenone	98-86-2	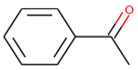	1	Training	1	Training	CC(=O)c1ccccc1
1,3 Indanedione	606-23-5	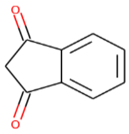			0	Training	O=C1CC(=O)c2ccccc21
3,4,5 Trifluorobenzyl alcohol	220227-37-2	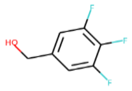			0	Training	OCc1cc(F)c(F)c(F)c1
Terephthalaldehyde	623-27-8	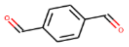			0	Test	O=Cc1ccc(C=O)cc1

Isophthalaldehyde	626-19-7	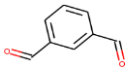	0	Training	O=Cc1cccc(C=O)c1
salicylic acid	63-36-5	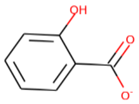	0	Training	O=C([O-])c1ccccc1O
2,4,5 Trifluorobenzyl alcohol	144284-25-3	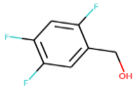	0	Training	OCc1cc(F)c(F)cc1F
1,3 benzenedimethanol	626-18-6	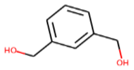	0	Training	OCc1cccc(CO)c1
2-Methoxybenzoic acid		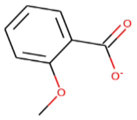	0	Training	COc1ccccc1C(=O)[O-]
1-Naphthaldehyde	66-77-3	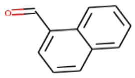	0	Training	O=Cc1cccc2ccccc12
3,4 Difluorobenzyl alcohol	85118-05-4	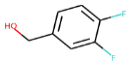	0	Training	OCc1ccc(F)c(F)c1
2,3,4 Trifluorobenzyl alcohol	144284-24-2	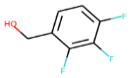	0	Training	OCc1ccc(F)c(F)c1F
3,5 Difluorobenzyl alcohol	79538-20-8	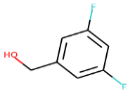	0	Test	OCc1cc(F)cc(F)c1
2,4 Difluorobenzyl alcohol	56456-47-4	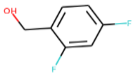	0	Training	OCc1ccc(F)cc1F
Phenylglyoxal monohydrate	1074-12-0	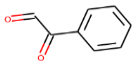	0	Training	O=CC(=O)c1ccccc1
3,5 Difluorobenzaldehyde	32085-88-4	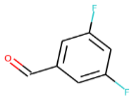	0	Training	O=Cc1cc(F)cc(F)c1
4-Fluorobenzyl alcohol	459-56-3	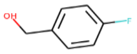	0	Training	OCc1ccc(F)cc1
2,5 Difluorobenzyl alcohol	75853-20-2	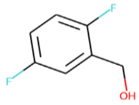	0	Training	OCc1cc(F)ccc1F

3, Fluorobenzyl alcohol	456-47-3	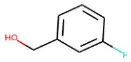	0	Training	OCc1cccc(F)c1
3,4 Difluorobenzaldehyde	34036-07-2	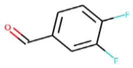	0	Training	O=Cc1ccc(F)c(F)c1
2,3 Difluorobenzyl alcohol	75853-18-8	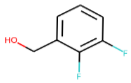	0	Training	OCc1cccc(F)c1F
4-Fluorobenzaldehyde	459-57-4	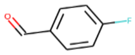	1	Test	O=Cc1ccc(F)cc1
2,4 Difluorobenzaldehyde	1550-35-2	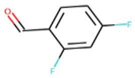	1	Training	O=Cc1ccc(F)cc1F
2,5 Difluorobenzaldehyde	2646-90-4	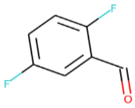	1	Training	O=Cc1cc(F)ccc1F
2,3 Difluorobenzaldehyde	2646-91-5	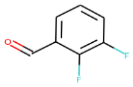	1	Training	O=Cc1cccc(F)c1F
2',4' Difluoroacetophenone	364-83-0	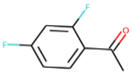	1	Training	CC(=O)c1ccc(F)cc1F
3- Fluorobenzaldehyde	456-48-4	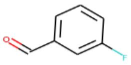	1	Training	O=Cc1cccc(F)c1
2-Fluorobenzyl alcohol	446-51-5	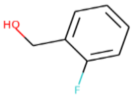	1	Training	OCc1ccccc1F
2,6 Difluorobenzaldehyde	437-81-0	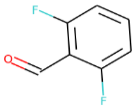	1	Training	O=Cc1c(F)cccc1F
4' Fluoroacetophenone	403-42-9	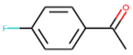	1	Test	CC(=O)c1ccc(F)cc1
P-tolualdehyde	104-87-0	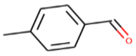	1	Training	Cc1ccc(C=O)cc1
2-Fluorobenzaldehyde	446-52-6	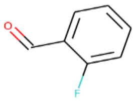	1	Training	O=Cc1ccccc1F

1-(2-fluorophenyl)ethanone	445-27-2	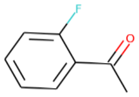	1	Training	CC(=O)c1ccccc1F
