# Supplementary material for: Reverse chemical ecology in a moth: machine learning on odorant receptors identifies new behaviorally active agonists
Source: Cell Mol Life Sci. 2021 Aug 27;78(19-20):6593–603. doi: 10.1007/s00018-021-03919-2 (PMC8558168; doi:10.1007/s00018-021-03919-2)
Supplement: Supplementary file 3 — Supplementary file3 (PDF 1117 KB) [file 18_2021_3919_MOESM3_ESM.pdf]

Reverse chemical ecology in a moth: machine learning on odorant receptors identifies new behaviorally active agonists

CMLS

Gabriela Caballero-Vidal[1§¤], Cédric Bouysset[2§], Jérémy Gévar[1], Hayat Mbouzid[1], Céline Nara[1], Julie Delaroche[1], Jérôme Golebiowski[2,3], Nicolas Montagné[1*], Sébastien Fiorucci[2*], & Emmanuelle Jacquin-Joly[1*]

[1] INRAE, Sorbonne Université, CNRS, IRD, UPEC, Université de Paris, Institute of Ecology and Environmental Sciences of Paris, Versailles 78000, France
[2] Université Côte d'Azur, CNRS, Institut de Chimie de Nice UMR7272, Nice 06000, France
[3] Department of Brain and Cognitive Sciences, Daegu Gyeongbuk Institute of Science and Technology, Daegu 711-873, South Korea
[¤] present address: Disease Vector Group, Chemical Ecology, Department of Plant Protection Biology, Swedish University of Agricultural Sciences, Alnarp, Sweden
Max Planck Centre Next Generation Chemical Ecology, Uppsala, Sweden

[§]both authors contributed equally to the work

*Corresponding authors:
Emmanuelle Jacquin-Joly
emmanuelle.joly@inrae.fr

Sébastien Fiorucci
sebastien.fiorucci@univ-cotedazur.fr

Nicolas Montagné
nicolas.montagne@sorbonne-universite.fr

Online Resource 3. Molecules predicted as agonists (A) or non-agonists (N) within the applicability domain of SlitOR24 (RandomForest model: RF) and SlitOR25 (former Support Vector Machine model from (20): SVM, and new k-nearest neighbor model: kNN) models. Molecules that were further experimentally tested on both ORs are indicated (Yes/No) as well as their activity on the corresponding OR (Yes/No).

Molecules	CAS	SlitOR24 prediction	SlitOR25 prediction	Previous SlitOR25 prediction	Molecules tested in SSR	Activity on SlitOR24	Activity on SlitOR25
3-heptanone	106-35-4	A	A	A	Yes	Yes	Yes
anisole	100-66-3	A	A	A	Yes	Yes	Yes
1-hexen-3-ol	4798-44-1	A	A	A	Yes	Yes	Yes
ethyl benzene	100-41-4	A	A	A	Yes	No	Yes
(*E*)-3-hexen-1-ol	928-97-2	A	A	A	Yes	Yes	Yes
(*Z*)-2-hexen-1-ol	928-94-9	A	A	A	Yes	Yes	Yes
2-heptanol	543-49-7	A	A	A	Yes	Yes	Yes
1-pentanol	71-41-0	A	A	A	Yes	Yes	Yes
2-heptanone	110-43-0	A	A	A	Yes	Yes	Yes
2-hexanone	591-78-6	A	A	A	Yes	Yes	Yes
2-hexanol	626-93-7	A	A	A	Yes	Yes	Yes
hexanal	66-25-1	A	A	A	Yes	Yes	Yes
benzyl cyanide	140-29-4	A	A	A	Yes	Yes	Yes
heptanal	111-71-7	A	A	A	Yes	Yes	Yes
4-methylanisole	104-93-8	N	A	A	Yes	Yes	Yes
2-phenylethanol	60-12-8	N	A	A	Yes	Yes	Yes
ethyl valerate	539-82-2	N	A	A	Yes	No	Yes
4-ethylphenol	123-07-9	N	A	A	Yes	Yes	Yes
2-methylbutyl acetate	624-41-9	N	A	A	Yes	Yes	Yes
propyl butyrate	105-66-8	N	A	A	Yes	No	Yes
2-butyl acetate	105-45-4	A	A	N	Yes	Yes	No
furfuryl alcohol	98-00-0	A	A	N	Yes	Yes	No
butyl formate	592-84-7	A	A	N	Yes	Yes	No
methyl 2-methylbutyrate	868-57-5	A	A	N	Yes	No	No
1-penten-4-ol	625-31-0	A	A	N	Yes	Yes	No
3-hexanone	589-38-8	A	A	N	Yes	Yes	Yes
ethyl butyrate	105-54-4	A	A	N	Yes	Yes	No
2-methyl-1-butanol	137-32-6	A	A	N	Yes	Yes	No
2-pentanol	6032-29-7	A	A	N	Yes	Yes	No
2-methyl-3-hexanol	617-29-8	A	A	N	Yes	Yes	No
propyl acetate	109-60-4	A	A	N	Yes	Yes	No
butyl isothiocyanate	592-82-5	A	A	N	Yes	Yes	No
propyl propionate	106-36-5	A	A	N	Yes	Yes	Yes
4-methyl-2-pentanol	108-11-2	A	N	N	Yes	Yes	No
2-methoxy-4-vinylphenol	7786-61-0	N	A	N	No		
isopropyl acetate	108-21-4	N	A	N	No		
1-butanol	71-36-3	N	A	N	No		
allyl acetate	591-87-7	N	A	N	No		
L-carvone	6485-40-1	N	A	N	No		
D-carvone	2244-16-8	N	A	N	No		
isoamyl acetate	123-92-2	N	A	N	No		
isobutyl acetate	110-19-0	N	A	N	No		
ethyl (S)-(-)-lactate	687-47-8	N	A	N	No		
4-butyrolactone	96-48-0	N	A	N	No		

2,3-butanediol	513-85-9	N	A	N	No		
ethyl 3-hydroxybutyrate	5405-41-4	N	A	N	No		
2-pentanone	107-87-9	N	A	N	No		
p-xylene	106-42-3	N	A	N	No		
methionol	505-10-2	N	A	N	No		
ethyl isovalerate	108-64-5	N	A	N	No		
methyl (S)-(-)-lactate	27871-49-4	N	A	N	No		
3-pentanone	96-22-0	N	A	N	No		
ethyl 3-methylcrotonate	638-10-8	N	A	N	No		
ethyl propionate	105-37-3	N	A	N	No		
isopropyl butyrate	638-11-9	N	A	N	No		
ethyl tiglate	5837-78-5	N	A	N	No		
4-methylvaleric acid	646-07-1	N	A	N	No		
isobutyl isobutyrate	97-85-8	N	A	N	No		
propyl isothiocyanate	628-30-8	N	A	N	No		
2-methylbutyric acid	116-53-0	N	A	N	No		
ethyl isobutyrate	97-62-1	N	A	N	No		
p-Tolyl acetate	140-39-6	N	A	N	No		
ethyl 2-methylbutyrate	7452-79-1	N	A	N	No		
3-methyl-3-pentanol	77-74-7	N	A	N	No		
ethyl benzoate	93-89-0	N	A	N	No		
methyl isobutyrate	547-63-7	N	A	N	No		
2,3-dimethoxytoluene	4463-33-6	N	A	N	No		
3-methyl-2-butanol	598-75-4	N	A	N	No		
D-limonene	5989-27-5	N	N	N	Yes	No	No
gamma nonalactone	104-61-0	N	N	N	Yes	No	No
(-)-menthone	14073-97-3	N	N	N	Yes	No	No
butyl propionate	590-01-2	N	N	N	Yes	No	No
eucalyptol	470-82-6	N	N	N	Yes	No	No

Online Resource 3. Molecules predicted as agonists (A) or non-agonists (N) within the applicability domain of SlitOR24 (RandomForest model: RF) and SlitOR25 (former Support Vector Machine model from (20): SVM, and new k-nearest neighbor model: kNN) models. Molecules that were further experimentally tested on both ORs are indicated (Yes/No) as well as their activity on the corresponding OR (Yes/No).
