# Supplementary material for: Reverse chemical ecology in a moth: machine learning on odorant receptors identifies new behaviorally active agonists
Source: Cell Mol Life Sci. 2021 Aug 27;78(19-20):6593–603. doi: 10.1007/s00018-021-03919-2 (PMC8558168; doi:10.1007/s00018-021-03919-2)
Supplement: Supplementary file 4 — Supplementary file4 (DOCX 27 KB) [file 18_2021_3919_MOESM4_ESM.docx]

**Reverse chemical ecology in a moth: machine learning on odorant receptors identifies new behaviorally active agonists**

**CMLS**

Gabriela Caballero-Vidal^1§¤^, Cédric Bouysset^2§^, Jérémy Gévar^1^, Hayat Mbouzid^1^, Céline Nara^1^, Julie Delaroche^1^, Jérôme Golebiowski^2,3^, Nicolas Montagné^1*^, Sébastien Fiorucci^2*^, & Emmanuelle Jacquin-Joly^1*^

^1^ INRAE, Sorbonne Université, CNRS, IRD, UPEC, Université de Paris, Institute of Ecology and Environmental Sciences of Paris, Versailles 78000, France

^2^ Université Côte d’Azur, CNRS, Institut de Chimie de Nice UMR7272, Nice 06000, France

^3^ Department of Brain and Cognitive Sciences, Daegu Gyeongbuk Institute of Science and Technology, Daegu 711-873, South Korea

^¤^ present address: Disease Vector Group, Chemical Ecology, Department of Plant Protection Biology, Swedish University of Agricultural Sciences, Alnarp, Sweden

Max Planck Centre Next Generation Chemical Ecology, Uppsala, Sweden

^§^both authors contributed equally to the work

*Corresponding authors:

**Emmanuelle Jacquin-Joly**

emmanuelle.joly@inrae.fr

**Sébastien Fiorucci**

sebastien.fiorucci@univ-cotedazur.fr

**Nicolas Montagné**

nicolas.montagne@sorbonne-universite.fr

**Online Resource 4.** Performance of the QSAR models when changing the initial test compounds used in the sphere-exclusion algorithm to obtain the training and test datasets. Only the first five splits that had the same activity distribution as in the split used for the final model were investigated.

|  | **Split #** | **Dataset** | **TP** | **TN** | **FP** | **FN** | **Accuracy** | **Precision** | **Recall** | **FPR** | **MCC** | **AUROC** |
| --- | --- | --- | --- | --- | --- | --- | --- | --- | --- | --- | --- | --- |
| **SlitOR24** | **1** | **LOO** | 4 | 29 | 2 | 4 | 0.85 | 0.67 | 0.50 | 0.06 | 0.49 | 0.93 |
|  |  | **Training** | 7 | 31 | 0 | 1 | 0.97 | 1.00 | 0.88 | 0.00 | 0.92 | 1.00 |
|  |  | **Test** | 0 | 10 | 0 | 2 | 0.83 | NA | 0.00 | 0.00 | NA | 0.50 |
|  | **2** | **LOO** | 3 | 29 | 2 | 5 | 0.82 | 0.60 | 0.38 | 0.06 | 0.38 | 0.80 |
|  |  | **Training** | 7 | 31 | 0 | 1 | 0.97 | 1.00 | 0.88 | 0.00 | 0.92 | 1.00 |
|  |  | **Test** | 1 | 10 | 0 | 1 | 0.92 | 1.00 | 0.50 | 0.00 | 0.67 | 0.95 |
|  | **3** | **LOO** | 3 | 29 | 2 | 5 | 0.82 | 0.60 | 0.38 | 0.06 | 0.38 | 0.80 |
|  |  | **Training** | 7 | 31 | 0 | 1 | 0.97 | 1.00 | 0.88 | 0.00 | 0.92 | 1.00 |
|  |  | **Test** | 1 | 10 | 0 | 1 | 0.92 | 1.00 | 0.50 | 0.00 | 0.67 | 0.95 |
|  | **4** | **LOO** | 4 | 29 | 2 | 4 | 0.85 | 0.67 | 0.50 | 0.06 | 0.49 | 0.83 |
|  |  | **Training** | 7 | 31 | 0 | 1 | 0.97 | 1.00 | 0.88 | 0.00 | 0.92 | 1.00 |
|  |  | **Test** | 0 | 10 | 0 | 2 | 0.83 | NA | 0.00 | 0.00 | NA | 0.85 |
|  | **5** | **LOO** | 3 | 29 | 2 | 5 | 0.82 | 0.60 | 0.38 | 0.06 | 0.38 | 0.80 |
|  |  | **Training** | 7 | 31 | 0 | 1 | 0.97 | 1.00 | 0.88 | 0.00 | 0.92 | 1.00 |
|  |  | **Test** | 1 | 10 | 0 | 1 | 0.92 | 1.00 | 0.50 | 0.00 | 0.67 | 0.95 |
|  | **Model** | **LOO** | 4 | 29 | 2 | 4 | 0.85 | 0.67 | 0.50 | 0.06 | 0.49 | 0.83 |
|  |  | **Training** | 7 | 31 | 0 | 1 | 0.97 | 1.00 | 0.88 | 0.00 | 0.92 | 0.99 |
|  |  | **Test** | 1 | 9 | 1 | 1 | 0.83 | 0.50 | 0.50 | 0.10 | 0.40 | 0.80 |
| **SlitOR25** | **1** | **LOO** | 14 | 38 | 8 | 4 | 0.81 | 0.64 | 0.78 | 0.17 | 0.57 | 0.87 |
|  |  | **Training** | 18 | 46 | 0 | 0 | 1.00 | 1.00 | 1.00 | 0.00 | 1.00 | 1.00 |
|  |  | **Test** | 6 | 8 | 4 | 1 | 0.74 | 0.60 | 0.86 | 0.33 | 0.51 | 0.77 |
|  | **2** | **LOO** | 15 | 36 | 10 | 3 | 0.80 | 0.60 | 0.83 | 0.22 | 0.57 | 0.89 |
|  |  | **Training** | 18 | 46 | 0 | 0 | 1.00 | 1.00 | 1.00 | 0.00 | 1.00 | 1.00 |
|  |  | **Test** | 6 | 9 | 3 | 1 | 0.79 | 0.67 | 0.86 | 0.25 | 0.59 | 0.81 |
|  | **3** | **LOO** | 15 | 36 | 10 | 3 | 0.80 | 0.60 | 0.83 | 0.22 | 0.57 | 0.89 |
|  |  | **Training** | 18 | 46 | 0 | 0 | 1.00 | 1.00 | 1.00 | 0.00 | 1.00 | 1.00 |
|  |  | **Test** | 6 | 9 | 3 | 1 | 0.79 | 0.67 | 0.86 | 0.25 | 0.59 | 0.81 |
|  | **4** | **LOO** | 11 | 39 | 7 | 7 | 0.78 | 0.61 | 0.61 | 0.15 | 0.46 | 0.89 |
|  |  | **Training** | 16 | 41 | 5 | 2 | 0.89 | 0.76 | 0.89 | 0.11 | 0.75 | 0.96 |
|  |  | **Test** | 6 | 6 | 6 | 1 | 0.63 | 0.50 | 0.86 | 0.50 | 0.36 | 0.69 |
|  | **5** | **LOO** | 15 | 38 | 8 | 3 | 0.83 | 0.65 | 0.83 | 0.17 | 0.62 | 0.89 |
|  |  | **Training** | 16 | 39 | 7 | 2 | 0.86 | 0.70 | 0.89 | 0.15 | 0.69 | 0.94 |
|  |  | **Test** | 7 | 6 | 6 | 0 | 0.68 | 0.54 | 1.00 | 0.50 | 0.52 | 0.85 |
|  | **Model** | **LOO** | 15 | 34 | 12 | 3 | 0.77 | 0.56 | 0.83 | 0.26 | 0.52 | 0.84 |
|  |  | **Training** | 18 | 46 | 0 | 0 | 1.00 | 1.00 | 1.00 | 0.00 | 1.00 | 1.00 |
|  |  | **Test** | 5 | 10 | 2 | 2 | 0.79 | 0.71 | 0.71 | 0.17 | 0.55 | 0.89 |
