# Supplementary material for: Reverse chemical ecology in a moth: machine learning on odorant receptors identifies new behaviorally active agonists
Source: Cell Mol Life Sci. 2021 Aug 27;78(19-20):6593–603. doi: 10.1007/s00018-021-03919-2 (PMC8558168; doi:10.1007/s00018-021-03919-2)
Supplement: Supplementary file 5 — Supplementary file5 (DOCX 16 KB) [file 18_2021_3919_MOESM5_ESM.docx]

**Reverse chemical ecology in a moth: machine learning on odorant receptors identifies new behaviorally active agonists**

**CMLS**

Gabriela Caballero-Vidal^1§¤^, Cédric Bouysset^2§^, Jérémy Gévar^1^, Hayat Mbouzid^1^, Céline Nara^1^, Julie Delaroche^1^, Jérôme Golebiowski^2,3^, Nicolas Montagné^1*^, Sébastien Fiorucci^2*^, & Emmanuelle Jacquin-Joly^1*^

^1^ INRAE, Sorbonne Université, CNRS, IRD, UPEC, Université de Paris, Institute of Ecology and Environmental Sciences of Paris, Versailles 78000, France

^2^ Université Côte d’Azur, CNRS, Institut de Chimie de Nice UMR7272, Nice 06000, France

^3^ Department of Brain and Cognitive Sciences, Daegu Gyeongbuk Institute of Science and Technology, Daegu 711-873, South Korea

^¤^ present address: Disease Vector Group, Chemical Ecology, Department of Plant Protection Biology, Swedish University of Agricultural Sciences, Alnarp, Sweden

Max Planck Centre Next Generation Chemical Ecology, Uppsala, Sweden

^§^both authors contributed equally to the work

*Corresponding authors:

**Emmanuelle Jacquin-Joly**

emmanuelle.joly@inrae.fr

**Sébastien Fiorucci**

sebastien.fiorucci@univ-cotedazur.fr

**Nicolas Montagné**

nicolas.montagne@sorbonne-universite.fr

**Online Resource 5**. Range of metrics values (min and max) for all splits investigated in Online Resource 1.

| **Target** | **Dataset** | **Accuracy** | **Precision** | **Recall** | **FPR** | **MCC** | **AUROC** |
| --- | --- | --- | --- | --- | --- | --- | --- |
| **SlitOR24** | LOO | 0.82–0.85 | 0.60–0.67 | 0.38–0.50 | 0.06 | 0.38–0.49 | 0.80–0.93 |
|  | Training | 0.97 | 1.00 | 0.88 | 0.00 | 0.92 | 1.00 |
|  | Test | 0.83–0.92 | NA–1.00 | 0.00–0.50 | 0.00–0.10 | NA–0.67 | 0.50–0.95 |
| **SlitOR25** | LOO | 0.77–0.83 | 0.56–0.65 | 0.61–0.83 | 0.10–0.26 | 0.46–0.62 | 0.84–0.89 |
|  | Training | 0.86–1.00 | 0.70–1.00 | 0.89–1.00 | 0.00–0.15 | 0.69–1.00 | 0.94–1.00 |
|  | Test | 0.63–0.79 | 0.50–0.71 | 0.71–1.00 | 0.17–0.50 | 0.36–0.59 | 0.69–0.89 |
