# Supplementary material for: Reverse chemical ecology in a moth: machine learning on odorant receptors identifies new behaviorally active agonists
Source: Cell Mol Life Sci. 2021 Aug 27;78(19-20):6593–603. doi: 10.1007/s00018-021-03919-2 (PMC8558168; doi:10.1007/s00018-021-03919-2)
Supplement: Supplementary file 6 — Supplementary file6 (DOCX 15 KB) [file 18_2021_3919_MOESM6_ESM.docx]

**Reverse chemical ecology in a moth: machine learning on odorant receptors identifies new behaviorally active agonists**

**CMLS**

Gabriela Caballero-Vidal^1§¤^, Cédric Bouysset^2§^, Jérémy Gévar^1^, Hayat Mbouzid^1^, Céline Nara^1^, Julie Delaroche^1^, Jérôme Golebiowski^2,3^, Nicolas Montagné^1*^, Sébastien Fiorucci^2*^, & Emmanuelle Jacquin-Joly^1*^

^1^ INRAE, Sorbonne Université, CNRS, IRD, UPEC, Université de Paris, Institute of Ecology and Environmental Sciences of Paris, Versailles 78000, France

^2^ Université Côte d’Azur, CNRS, Institut de Chimie de Nice UMR7272, Nice 06000, France

^3^ Department of Brain and Cognitive Sciences, Daegu Gyeongbuk Institute of Science and Technology, Daegu 711-873, South Korea

^¤^ present address: Disease Vector Group, Chemical Ecology, Department of Plant Protection Biology, Swedish University of Agricultural Sciences, Alnarp, Sweden

Max Planck Centre Next Generation Chemical Ecology, Uppsala, Sweden

^§^both authors contributed equally to the work

*Corresponding authors:

**Emmanuelle Jacquin-Joly**

emmanuelle.joly@inrae.fr

**Sébastien Fiorucci**

sebastien.fiorucci@univ-cotedazur.fr

**Nicolas Montagné**

nicolas.montagne@sorbonne-universite.fr

**Online Resource 6.** Performance of the QSAR models on the new experimental data.

| **Target** | **TP** | **TN** | **FP** | **FN** | **Accuracy** | **Precision** | **Recall** | **FPR** | **MCC** |
| --- | --- | --- | --- | --- | --- | --- | --- | --- | --- |
| **SlitOR24** | 26 | 6 | 2 | 5 | 0.82 | 0.93 | 0.84 | 0.25 | 0.53 |
| **SlitOR25** | 22 | 6 | 11 | 0 | 0.72 | 0.67 | 1.00 | 0.65 | 0.49 |
