# Supplementary material for: Reverse chemical ecology in a moth: machine learning on odorant receptors identifies new behaviorally active agonists
Source: Cell Mol Life Sci. 2021 Aug 27;78(19-20):6593–603. doi: 10.1007/s00018-021-03919-2 (PMC8558168; doi:10.1007/s00018-021-03919-2)
Supplement: Supplementary file 7 — Supplementary file7 (DOCX 16 KB) [file 18_2021_3919_MOESM7_ESM.docx]

**Reverse chemical ecology in a moth: machine learning on odorant receptors identifies new behaviorally active agonists**

**CMLS**

Gabriela Caballero-Vidal^1§¤^, Cédric Bouysset^2§^, Jérémy Gévar^1^, Hayat Mbouzid^1^, Céline Nara^1^, Julie Delaroche^1^, Jérôme Golebiowski^2,3^, Nicolas Montagné^1*^, Sébastien Fiorucci^2*^, & Emmanuelle Jacquin-Joly^1*^

^1^ INRAE, Sorbonne Université, CNRS, IRD, UPEC, Université de Paris, Institute of Ecology and Environmental Sciences of Paris, Versailles 78000, France

^2^ Université Côte d’Azur, CNRS, Institut de Chimie de Nice UMR7272, Nice 06000, France

^3^ Department of Brain and Cognitive Sciences, Daegu Gyeongbuk Institute of Science and Technology, Daegu 711-873, South Korea

^¤^ present address: Disease Vector Group, Chemical Ecology, Department of Plant Protection Biology, Swedish University of Agricultural Sciences, Alnarp, Sweden

Max Planck Centre Next Generation Chemical Ecology, Uppsala, Sweden

^§^both authors contributed equally to the work

*Corresponding authors:

**Emmanuelle Jacquin-Joly**

emmanuelle.joly@inrae.fr

**Sébastien Fiorucci**

sebastien.fiorucci@univ-cotedazur.fr

**Nicolas Montagné**

nicolas.montagne@sorbonne-universite.fr

**Online Resource 7.** List of putative SlitOR24 and 25 residues corresponding to *Machilis hrabei* (MhraOR5) odorant-binding site according to ClustalO and MAFFT multiple sequence alignments (MSA). The MSA have been performed on the EMBL-EBI webserver.

| MhraOR5 | SlitOR24 | | SlitOR25 | |
| --- | --- | --- | --- | --- |
|  | ClustalO | MAFFT | ClustalO | MAFFT |
| V88 | V88 | V88 | V88 | V88 |
| Y91 | I91 | I91 | L91 | L91 |
| F92 | H92 | H92 | Q92 | Q92 |
| S151 | T153 | T153 | T153 | T153 |
| G154 | V156 | V156 | A156 | A156 |
| W158 | Y160 | Y160 | F160 | F160 |
| M209 | I195 | F197 | I195 | F197 |
| I213 | Y199 | S201 | Y199 | S201 |
| Y380 | Y322 | Y322 | F322 | F322 |
| Y383 | Y325 | Y325 | Y325 | Y325 |
